# Alginate Hydrogels Reinforced by Dehydration under Stress—Application to a Soft Magnetic Actuator

**DOI:** 10.3390/gels9010039

**Published:** 2023-01-03

**Authors:** Alberto Leon-Cecilla, Francisco J. Vazquez-Perez, Cristina Gila-Vilchez, Luis Álvarez de Cienfuegos, Modesto T. Lopez-Lopez

**Affiliations:** 1 Departamento de Física Aplicada, Universidad de Granada, C. U. Fuentenueva, E-18071 Granada, Spain; 2 Instituto de Investigación Biosanitaria ibs.Granada, Avda. de Madrid 15, E-18012 Granada, Spain; 3 Unidad de Excelencia Química Aplicada a Biomedicina y Medioambiente, Departamento de Química Orgánica, Universidad de Granada, C. U. Fuentenueva, E-18071 Granada, Spain

**Keywords:** polymer hydrogels, alginate, mechanical properties, soft magnetic actuator

## Abstract

We investigated the effect of partial dehydration under mechanical stress in the properties of alginate hydrogels. For this aim, we characterized the mechanical properties of the hydrogels under tensile and shear stress, as well as their swelling behavior, macroscopic appearance, and microscopic structure. We found that the processes of dehydration under a mechanical stress were irreversible with fully rehydration being impossible. What is more, these processes gave rise to an enhancement of the mechanical robustness of the hydrogels beyond the effect due to the increase in polymer concentration caused by dehydration. Finally, we analyzed the applicability of these results to alginate-based magnetic hydrogel grippers that bended in response to an applied magnetic field. Remarkably, our study demonstrated that the dehydration of the magnetic hydrogels under compression facilitated their bending response.

## 1. Introduction

During the last few decades, there has been an increasing interest in developing novel polymeric materials for biomedical [1,2,3,4,5,6] and technological applications [7,8,9,10,11,12]. Among the different polymeric materials, hydrogels have received special attention due to the possibilities offered by their softness, porosity, high water content, biocompatibility, and modifiable mechanical properties. Indeed, hydrogels stand out versus other polymeric materials mainly because they have an elevated swelling capacity and resemble living tissues better than any other synthetic biomaterial. These facts make them ideal candidates for many applications [13,14,15,16,17,18,19,20], such as soft actuators [21,22,23,24], drug delivery [25,26], cell culture and encapsulation [27], wound dressing [28,29], or the production of scaffolds for tissue engineering [30,31,32,33,34]. As hydrogels are defined as three-dimensional cross-linked hydrophilic polymeric networks swollen by a continuous aqueous medium [13], the swelling degree will be decisive not only in terms of their inner structure, but also in terms of their mechanical properties. It is well known that, at equilibrium, hydrogels can reach a water content up to 99% in volume. However, a subsequent dehydration process will lead to a decrease in the swelling degree of the hydrogel and, therefore, a corresponding increase in the polymer concentration. This fact, along with the resultant polymer–polymer interactions, may have a dramatic impact on the mechanical properties of these materials.

As the porosity and microstructure of the hydrogels are crucial characteristics in their application as extracellular matrices [35], the effect of the dehydration processes has been studied from the point of view of the changes in their inner structure [36,37]. For example, most synthesized hydrogels generally have an isotropic internal structure [38], while natural tissues such as cartilage [39,40], muscle [41,42], and skin [43,44] show anisotropic and well-defined hierarchical structures [45,46,47,48]. Indeed, anisotropy often plays an essential role in many natural processes and mechanisms, including mass transport, surface lubrication, and force generation. With nature being a source of inspiration for many advanced materials and applications, it is not surprising that anisotropic hydrogels are also receiving increasing interest in the field of biomimetics [49]. In line with this interest, different strategies for the preparation of anisotropic hydrogels have emerged in recent years. The most common procedures to obtain hydrogels with an internal anisotropic structure involve the application of directional stimuli such as gradients of temperature or ionic concentration [50]. Furthermore, the inclusion of magnetic particles into the hydrogel formulation makes it possible to obtain an anisotropic internal structuring through the application of external electric or magnetic fields [51,52,53,54,55]. Nevertheless, these strategies imply technical difficulties as far as a biocompatible and suitable design is concerned. Thus, the use of dehydration processes combined with mechanical deformations is widely proposed as the simplest and versatile way to obtain anisotropic hydrogels [36,37,50,56,57,58,59,60,61]. In a previous work [36], the authors used a method based on dehydration under tensile stress to fabricate anisotropic hydrogels with aligned fibrous structures, which resulted in enhanced values of the Young’s modulus with respect to gels dehydrated without subjecting them to stress. Similarly, in another work [37], we used compression as a method to induce the alignment of the polymeric fibers in parallel layers, reporting an improvement in the mechanical properties with respect to non-dehydrated (non-compressed) samples, although the question arises as to whether this improvement was due solely to the reduction in water caused by compression. However, despite the several research works that studied the effects of dehydration on the resultant microstructure of the hydrogels, their consequences on the mechanical properties of these materials are usually undervalued and misunderstood. Thus, a lack of complete rheological studies becomes apparent when it comes to focusing on the role of dehydration in the mechanical properties of hydrogels and the connection with the changes at the microscopic level. Furthermore, the potential effect of hydrogel dehydration on certain applications of hydrogels, such as soft actuators and robots with medical applications, is largely unknown. 

In connection to what has been discussed above, the main objective of this work is to carry out a comprehensive analysis of the effect of partial dehydration induced by mechanical stress (both tensile and compressive) on the mechanical properties of physical polymer hydrogels, and to connect these changes with modifications in the polymer arrangement at a microscopic level. What is more, we aim to analyze if these changes can make a substantial improvement on the response of soft magnetic actuators based on polymer hydrogels. For these aims, we chose alginate as the polymer material for the preparation of the hydrogels. Alginate, a polysaccharide extracted from brown algae, is marked by its biocompatibility, non-toxicity, abundance in source, and low cost [14]. Furthermore, the fact that alginate hydrogels are easily synthesized by adding a source of divalent cations to a solution of sodium alginate [31], makes this polymer an ideal candidate that prevails over others. In addition, we have shown in previous works that alginate hydrogels allow the incorporation of large amounts of magnetic particles, without negligible phase disruption [62,63]. In this work, we study alginate hydrogels, which are based on two sodium alginates of different molecular weights, dehydrated by two different methods (dehydration under tensile stress and dehydration under compressive stress). We analyze the influence of the dehydration process on the resulting properties of these hydrogels in terms of water loss, molecular weight of the alginates, mechanical properties, and microscopic structure. These materials could have interesting applications in tissue engineering due to their new overall performance after the dehydration. In addition, we investigate the effect that dehydration under compression has on the actuation response of an alginate-based magnetic hydrogel gripper that bends under a magnetic field with interesting future applications in medicine. Our results demonstrate that dehydration under a controlled stress positively affects the robustness of alginate hydrogels and facilitates the response of the magnetic hydrogel gripper to the applied magnetic field. Apparently, new bonds between polymer fibers which arise during the stress-controlled dehydration processes and the increase in polymer concentration seem to be responsible for this enhancement of the properties and actuation response of the hydrogels. Results from our work not only provide an unprecedented complete picture of the effect of dehydration under controlled stresses (both tensile and compressive) on the resulting mechanical properties of physical polymer hydrogels, but also analyze, for the first time, the consequences of these changes on the responsiveness of soft magnetic actuators. Interestingly, an enhancement of the magnetic responsiveness is found in connection with changes at the level of the microscopic arrangement of polymer strands.

## 2. Results and Discussion

### 2.1. Macroscopic Appearance, Dehydration, and Re-Swelling of the Alginate Hydrogels

Non-dehydrated alginate hydrogels kept the shape of the containers used for their preparation and had a homogeneous macroscopic appearance (Figure S1a). After the stress controlled dehydration processes (SCDP), only applied to C4.0AH100-0 and C4.0AH50-50 (see Section 4 for more details), they maintained similar macroscopic appearance and homogeneity. In all cases, hydrogels were translucent because of the high polymer concentration. Moreover, due to the partial loss of bulk water during the SCDP, the hydrogels shrank and decreased in volume (Figure S1b). The relatively high alginate concentration in conjunction with the high cross-linking density, resulted in robust alginate hydrogels that were easily manipulated, both before and after dehydration. However, there were differences in consistency between the AH100-0 and AH50-50, which will be reflected below in the results of their mechanical characterization.

As was previously said, the degree of dehydration was quantified with the alginate concentration and water lost after the SCDP. For this reason, the mass of original hydrogels and their final mass after the SCDP were measured to calculate the water lost and the final concentration of polymer. Table 1 shows the mean final alginate concentration of the hydrogels and the water lost after being dehydrated at three different times for each method of SCDP. As can be seen, we managed to obtain similar alginate final concentration by controlling and repeating the conditions of dehydration in each process. While the alginate concentration of original hydrogels was 4 wt.%, once dehydrated, we obtained a range of alginate concentrations between 4.41 ± 0.04 wt.% (wl = 9.8 ± 0.9 wt.%) and 6.2 ± 0.3 wt.% (wl = 37 ± 3 wt.%).

The original hydrogels were also dehydrated without any applied stress for 24 h at room temperature in contact with blotting paper and an alginate concentrations of 7.7 ± 0.3 wt.% (wl = 49.9 ± 2.2 wt.%) for the AH100-0 sample and 8.5 ± 0.9 wt.% (wl = 55 ± 6 wt.%) for the AH50-50 sample were obtained. Then, these hydrogels were rehydrated by immersing them in distilled water for an additional 24 h, achieving a partial recovery of the water lost. This resulted in alginate concentrations of 5.23 ± 0.17 wt.% (wl = 24.4 ± 0.3 wt.%) for the AH100-0 and 7.4 ± 0.7 wt.% (wl = 48 ± 6 wt.%) for the AH50-50. Nevertheless, the initial state of hydration was not recovered in any case, which points out the formation of new stable bonds among polymer chains during the dehydration, most likely, hydrogen bonds formed between the -OH groups of the alginate fibers during dehydration, as reported in a previous work for alginate hydrogels [36].

### 2.2. Mechanical Characterization of the Alginate Hydrogels

#### 2.2.1. Mechanical Characterization under Tensile Stress

**Figure 1:**
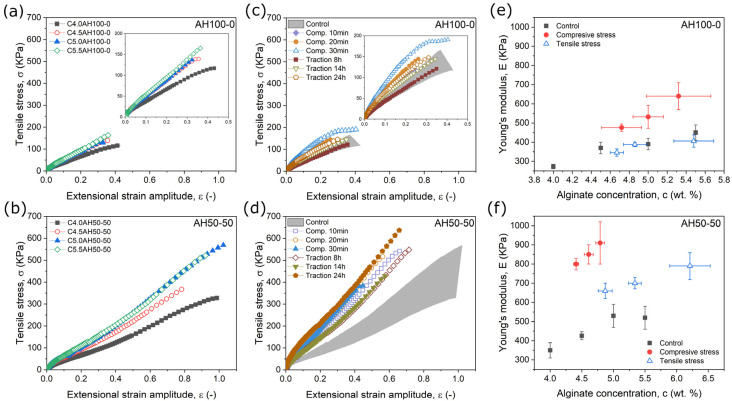
Results from uniaxial tensile characterization. From (**a**–**d**) strains vs. stress example curves for the different samples studied. (**a**,**b**) control samples of the two types of alginate hydrogels studied, the insets are the same data with a different scale. (**c**,**e**) strains vs. stress curves for each SCDP. Shaded bands represents the range of values for control samples. In (**e**,**f**) the values of Young’s modulus for each SCDPs and alginate concentration obtained after the dehydration are shown, as well as the values for control samples. Note that same scales are used in both axis.

From the uniaxial tensile characterization carried out for control samples (Figure 1a,b) it is clear that for a given value of the extensional strain amplitude, the corresponding tensile stress increased with the concentration of alginate, for both AH100-0 (Figure 1a) and AH50-50 (Figure 1b). Consequently, the Young’s modulus, defined as the slope of these curves, increased with the concentration of alginate in the control hydrogel samples (Figure 1c,d) as expected from the classical theory of the mechanical properties of networks and gels that predicts an increase of robustness with the volume concentration of polymer strands [64,65]. However, it is also clear that other factors had a role, since for example, the maximum stress and deformation at break were much larger for sample AH50-50 than for sample AH100-0 (compare Figure 1a,b). Nevertheless, the main aim of this work is to elucidate whether dehydration processes under stress have an effect on the mechanical properties of hydrogels beyond the increase of polymer concentration observed in Figure 1a,b. The answer is clear in Figure 1c,d, where it is observed that SCDP under compression gave rise to considerably higher values of the tensile stress for a given value of the extensional strain amplitude for both AH100-0 and AH50-50, in comparison with the control samples—remark the similar ranges of alginate concentrations for control samples and samples subjected to SCDP (Table 1). In the case of SCDP under tensile stress, there was a clear enhancement of tensile stress for a given strain amplitude for AH50-50, whereas no appreciable effect was observed for AH100-0, both with respect to control samples (Figure 1c,d). Correspondingly, the resulting Young’s modulus (Figure 1e,f) was in general enhanced more strongly for samples subjected to SCDP than for control samples, confirming the positive effect of dehydration and the consequent potential formation on new bonds between the alginate fibers of the hydrogels subjected to the SCDP. This effect was stronger in AH50-50, where a larger increase of Young’s modulus was observed regardless of the type of stress applied during SCDP and negligible for AH100-0 subjected to tensile stress during SCDP. 

From the curves of Figure 1, the stress and maximum deformation at the break of the samples were obtained. For these two magnitudes (Figure S2), statistically significant differences were observed between the two types of alginate hydrogels, with much higher values for AH50-50 than for AH100-0. Relating to the role of SDCP, a slight decrease in deformation at the fracture seemed to take place, whereas the stress at the break slightly increased for the AH100-0 samples and more strongly for the AH50-50 samples subjected to SCDP with respect to control samples (Figure S2a,b).

#### 2.2.2. Mechanical Characterization under Oscillatory Shear Stress

**Figure 2:**
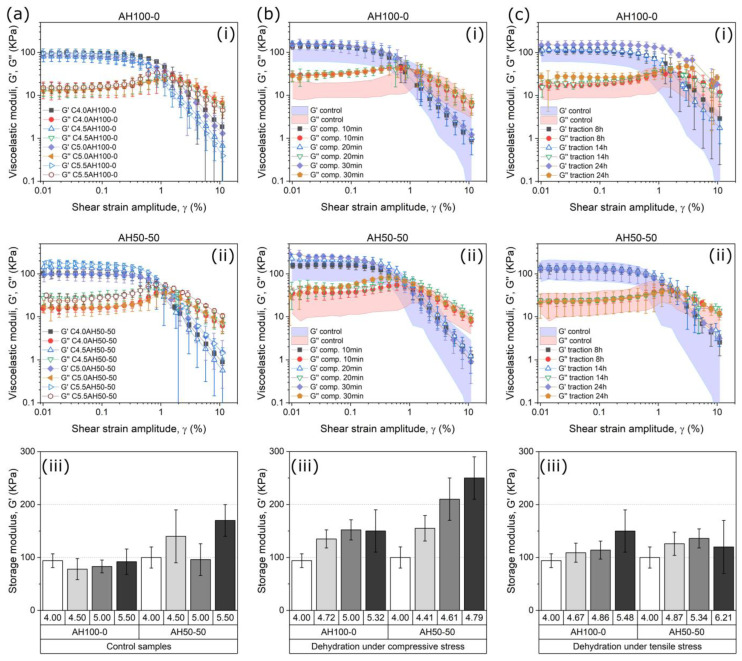
Results from the rheological characterization under oscillatory shear. The data in each column is related to the stress applied during the dehydration, (**a**) control samples, (**b**) samples dehydrated under compressive stress and (**c**) samples dehydrated under tensile stress. The data in the first two rows are presented as a comparison between the curves obtained for (**i**) AH100-0 and (**ii**) AH50-50, and in the third row (**iii**) the values of the storage modulus G′ obtained from the previous curves are shown. The areas in (**b**(**i**)), (**b**(**ii**)), (**c**(**i**)) and (**c**(**ii**)) represent the ranges of values for control samples. Note that same scales are used in both axes.

The rheological characterization of the samples under oscillatory shear stress revealed solid-like viscoelastic behavior, characterized by higher values of the elastic modulus (*G*′) than the loss modulus (*G*″) at low values of shear strain (Figure 2 and Figure S3). Curves corresponding to amplitude sweeps (Figure 2 rows (i,ii)) show that both moduli presented an initial plateau zone that defined the linear viscoelastic region. After this plateau zone, the storage modulus undergoes a sharp decrease while the loss modulus shows a small peak also followed by a sharp decrease for larger deformations. This region, where the viscoelastic moduli values do not remain constant, is known as non-linear viscoelastic region. Within this zone, the inner structure of the hydrogel breaks, which causes a lower elasticity in the material, evidenced by smaller values of *G*′. The shear strain amplitude (γ_0_) at which the peak in *G*″ is observed is known as yielding point where the dissipation of energy is at a maximum, and it marks the onset of the destruction of the polymeric network [66]. Similarly to the Young’s modulus, it is expected that both *G*′ and *G*″ increase with the content of polymer in cross-linked samples [62,63], and it is indeed what is observed in general terms for control samples (Figure 2a), although differences are almost negligible and tendencies are not always clear, very likely due to small differences in alginate content. The effect of compression during the SCDP on the values of *G*′ and *G*″ of the final hydrogels was made clear (Figure 2b). As observed for both AH100-0 and AH50-50, compression during the SCDP resulted in considerable larger values of *G*′ and *G*″ as compared with control samples with alginate content within a similar range. A similar enhancement with respect to the control samples was obtained for the AH100-0 samples subjected to tensile stress during SCDP, whereas no significant differences were observed for the AH50-50 samples dehydrated under similar conditions (Figure 2c).

Finally, the results of frequency sweeps (Figure S4) demonstrated a slight increase of both *G*′ and *G*″ with frequency, although the lost tangent (tan *δ* = *G*″/*G*′) was maintained higher than 0.1 for the whole range of frequencies under study, which means that no relative changes of the viscous character with respect to the elastic character with frequency of oscillation were observed. Furthermore, no significant differences on the shape of the curves of *G*′ and *G*″ vs. frequency (mechanical spectra) were caused by the SCDP, pointing out that the rearrangement of the fibers did not change the nature of the main interaction between the polymer chains (electrostatic bonds), which is primarily responsible for the mechanical spectra of the material.

In summary, these results together with the ones from the mechanical characterization under tensile stress demonstrate a general trend of hydrogels to become stiffer, and they can’t be easily deformed when the alginate concentration is increased with an additional reinforcement when the samples were subjected to the stress-controlled dehydration processes. Our results also demonstrate a crucial role of the type of alginate on the mechanical properties of the samples.

### 2.3. Analysis of the Anisotropy in the Hydrogels

From results of previous sections, it is clear that changes in the mechanical properties of samples subjected to SCDP are not just a consequence of the increase in concentration of polymer but should also be due to the appearance of new bonds between polymer strands (very likely hydrogen bonds between the -OH groups of the alginate fibers). To further investigate the consequences of SCDP in the hydrogels, in this subsection, the potential anisotropy in the hydrogels is analyzed. 

**Figure 3:**
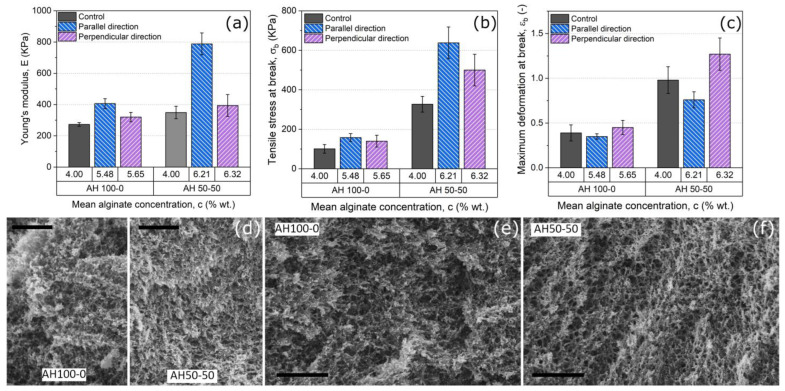
Young’s modulus, deformation and stress at break, and microscopic structure of the dehydrated alginate hydrogels under tensile stress. Data for control samples are included for comparison. In each graph (**a**–**c**) it is shown, from left to right, the value for control samples (C4.0AH100-0 and C4.0AH50-50), the value for samples measured under uniaxial tensile test parallel to the direction of the SCDP stress, and the value for the samples measured under uniaxial tensile test perpendicular to the direction of the SCDP stress. (**d**) SEM images of the polymeric network of AH100-0 and AH50-50 after their preparation (top view). (**e**,**f**) SEM image of the polymeric network of AH100-0 and AH50-50 (top view), respectively, after a tensile controlled dehydration process of 24 h duration. Scale bars correspond to 1 µm.

From the mechanical point of view, due to the planar geometry of our samples, only the hydrogels dehydrated under a tensile stress during SDCP could be characterized in two relevantly different perpendicular directions—parallel to the direction of the stress applied during SCDP and perpendicular to this direction (Figure S5a). From the results obtained (Figure 3), the existence of anisotropic mechanical behavior is clear. As observed, for both types of samples (AH100-0 and AH50-50) the values of the Young’s modulus for measurements under tensile stress applied in the parallel direction to the SCDP stress are higher than these obtained under tensile stress applied in the perpendicular direction to the SCDP stress. In comparison to the values of Young’s modulus (Figure 3a) for the control samples (C4.0AH100-0 and C4.0AH50-50), not subjected to SDCP, a statistically significant increase was only obtained for measurements under a tensile stress applied in the parallel direction to the SCDP stress. The stress at break (Figure 3b) also demonstrated larger values (although only statistically significant for the case of the AH50-50 sample) for measurements under tensile stress in parallel to the SCDP stress with respect to the measurements under tensile stress in the perpendicular direction. For this quantity (stress at break), values for measurements after SCDP in the case of the AH50-50 samples were considerably larger than this of the original (non-dehydrated) sample, no matter the direction of measurement (perpendicular or parallel to the direction of SCDP stress). Concerning the maximum deformation at the break (Figure 3c), the reverse tendency took place with the values for measurements under a tensile stress in the parallel direction to the SCDP stress being lower than the values for measurements under a tensile stress in the perpendicular direction, although differences observed in this case are only statistically significant for the AH50-50 samples. It should be noted that in general the effects and differences observed for the AH50-50 samples are much stronger than for the AH100-0 samples, demonstrating the relevant role of the length of the polymer strands on the changes caused by the SCDP.

All these results of mechanical characterization point to the appearance of new bonds between polymer chains of samples subjected to SCDP, as well as to potential changes at the level of microscopic arrangement of polymer strands. To elucidate on this point, we investigated the microscopic structure of the samples with SEM (Figure 3d–f). As observed, for control samples not subjected to SCDP, homogeneous and isotropic porous structures, without any fiber organization, were obtained (Figure 3d). For samples subjected to SCDP under tensile stress similar isotropic structures to control sample were observed in the case of AH100-0 (Figure 3e), whereas a clearer anisotropic structure consisting of parallel bands was observed in the case of AH50-50 (Figure 3f). These observations agree with the mechanical anisotropy discussed above, which is much more evident for the AH50-50 samples than for the AH100-0 samples (Figure 3a–c). Furthermore, note that similar results in terms of the existence of anisotropy to these samples subjected to SCDP under tensile stress were obtained for samples subjected to SCDP under compressive stress (Figure S6). 

As a general conclusion, it can be said that dehydration under stress gave rise to anisotropic and tougher hydrogels with respect to hydrogels containing similar concentrations of polymers but not subjected to dehydration. It is likely that the asymmetry induced by the SCDP resulted in hydrogen bonds formed between the -OH groups of the alginate fibers aligned in a privileged direction, which might be responsible for this reinforcement at the macroscopic level, as well as for the permanent changes in hydrogel shape, the appearance of mechanical, and microstructural anisotropy. We do not think that entanglement between polymer fibers is enhanced during SCDP because of the uniaxial nature of the hydrogel applied.

### 2.4. Role of SCDP in the Response of a Hydrogel-Based Magnetic Actuator

Application of hydrogels as functional blocks in actuators is a topic of increasing interest in the recent years [23]. Among the different stimuli to control actuation, magnetic fields represent one of the most attractive ones due to their easy use, remote and wireless control, prompt response, and safe penetration in biological environments [22]. For the magnetic actuation of hydrogels being possible, the combination of particles of magnetic materials with the polymer network is required, thus obtaining magnetic hydrogels [8]. In this section, we investigate if SCDP also have a positive effect on the magnetic actuation of hydrogels. Since according to results of previous sections, dehydration under compressive stress resulted in a stronger enhancement of the mechanical properties than dehydration under tensile stress (cf., Figure 1 and Figure 2), in the current section, we focus only on dehydration under compressive stress.

**Figure 4:**
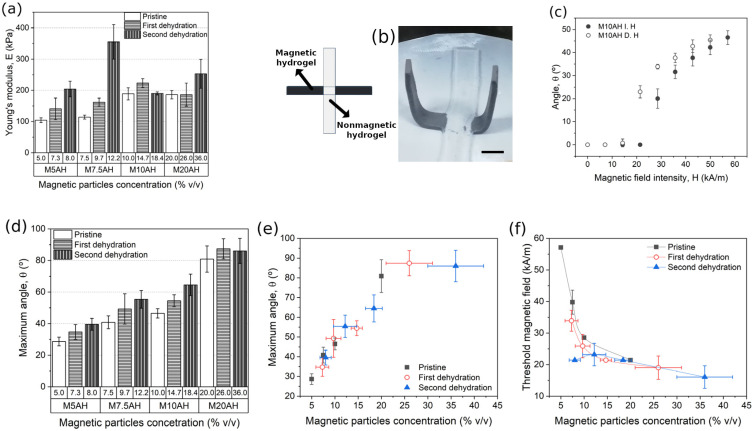
Mechanical response and actuation of the actuator. (**a**) Young’s modulus of the active part of the actuator before and after the two steps of dehydration under compressive stress. (**b**) Sketch and image of the actuator (on-state). Scale bar is 10 mm. (**c**) Angle as a function of the magnetic field intensity for pristine actuator with a particle concentration of 10% *v*/*v*. (**d**) Maximum angle reached by the actuator at a magnetic field intensity of 57 kA/m. (**e**) Maximum angle reached by the actuator at a field of 57 kA/m as function of the magnetic particle concentration. (**f**) Threshold magnetic field as function of the magnetic particle concentration of the actuator.

Concentrations of magnetic microparticles and alginate polymer after one and two cycles of dehydration under compression for magnetic hydrogels initially containing 1 wt.% of alginate are shown in Table 2. Similarly to the case of nonmagnetic hydrogels studied in the previous subsections, the dehydration under compression resulted in an overall trend of the Young’s modulus of magnetic hydrogels to increase, as the resulting alginate concentration also increased (Figure 4a). Under a magnetic field of high enough intensity, applied perpendicular to the plane of the actuator, the magnetic hydrogel beams bended in response to the magnetic torque appearing because of the misalignment between the magnetic beams and the direction of the applied magnetic field, resulting in a gripping movement (Figure 4b). We can quantify the response to the magnetic stimuli by the equilibrium angle of inclination of the magnetic beams of the actuator with respect to the horizontal (off-state) position (Figure S7). Equilibrium was reached as a balance between the magnetic torque favoring the alignment of the beams with the vertical direction and the torque due to the gravitational forces and the elastic forces, which opposed to this deformation. Rigorous analysis of the problem is very complicated, but we can have a rough idea by some simple approximations. First, on the base of the dipolar approximation, the magnetic torque, ${\vec{T}}_{m}$, acting on a body of magnetic moment $\vec{m}$ in a magnetic field of strength $\vec{H}$ is given by the following equation:

(1) $${\vec{T}}_{m}=\mu_{c}\vec{m}\times \vec{H}$$

$\mu_{c},$ being the magnetic permeability of the carrier medium. Note that $\vec{m}$ is an extensive quantity and thus it is the summation of the magnetic moments of the individual particles constituting the magnetic hydrogel. Concerning the elasticity of the hydrogel, according to Euler-Bernoulli beam theory the bending moment, $T_{b}$, due to elasticity, is given by the following equation:

(2) $$T_{b}=EI\frac{d^{2}u\left(x\right)}{dx^{2}},$$

*E* being the Young’s modulus, *I* the second moment of area of the beam, and $d^{2}u\left(x\right)/dx^{2}$ the curvature of the beam. Finally, the gravitational torque, $T_{g}$ is roughly given by the following:

(3) $$T_{g}=\frac{1}{2}Wlcos\;\theta ,$$

*W* being the weight of the beam, *l* its length and *θ* the angle of inclination of the beam with respect to the horizontal (off-state) position. Note that in the expression of the gravitational torque, the beam is approximated to a straight line (Figure S7b), whereas in the real case a curvature exists (Figure 4b) and the angle $\theta =asindu\left(x\right)/dx$ is not uniform (Figure 4b). Qualitatively, the magnetic torque increases with the concentration of particles in the magnetic hydrogel beam and with the strength of the applied magnetic field, whereas the bending moment increases with the Young’s modulus and the curvature of the beam. Regarding the gravitational torque, it is maximum in the off-state (horizontal position) and decreases as the angle of inclination increases.

Experiments demonstrated that there was a threshold value of the magnetic field strength required to actuate on the magnetic beams in actuation experiments (i.e., to obtain a nonzero equilibrium angle with respect to the off-state (horizontal) position) (Figure 4c and Figure S8). The existence of this threshold magnetic field strength agrees with the fact that the gravitational torque was nonzero (indeed, it was maximum) in the off-state. In equilibrium, the gravitational torque must be totally balanced by the magnetic torque as soon as the magnetic hydrogel beam detached from the horizontal surface used as support—note that the normal force exerted by the horizontal surface on the actuator that balanced the gravitational force in the absence of applied magnetic field disappeared as soon as the actuator was detached from this surface. For increasing magnetic field strengths above the threshold value, there was a progressive increase of the actuation angle with a tendency to saturate for the highest values of the field (Figure 4c and Figure S8). Subsequently, the actuation angle progressively decreased until zero as the applied magnetic field was decreased to zero from the maximum value. In all cases, the angle obtained increasing the magnetic field was less than the angle obtained decreasing the magnetic field, which indicates the existence of hysteresis. Furthermore, the threshold value of the magnetic field strength was lower when the field was decreased that when it was increased (Figure 4c and Figure S8).

In order to analyze the role of magnetic particle concentration and dehydration processes in the actuation behavior of the cross-shaped actuators, it is convenient to focus only on two quantities, the equilibrium angle at the maximum applied field (57 kA/m) and the threshold value of the magnetic field strength. In relation to the angle at the maximum applied field, it is clear that it increased with particle content, either for pristine (non-dehydrated) hydrogels or for dehydrated (one or two times) hydrogels (Figure 4d). This result is coherent with the expression of the magnetic torque, which is proportional to the magnetic moment of the magnetic hydrogel, should increase relatively to the elastic and gravitational ones as the concentration of magnetic particles increased. In addition, no effect of dehydration process, apart from the consequent effect due to the increase of particle concentration due to this process was observed since all experimental data for pristine and dehydrated samples fit on a single master curve vs. particle concentration (Figure 4e). Concerning the threshold magnetic field strength, it decreased with the concentration of magnetic particles, as expected from the proportionality of the magnetic torque with the magnetic moment of the hydrogel beam (Figure 4f). Moreover, curiously, the data of this quantity for the different samples (pristine, 1st dehydration, and 2nd dehydration) did not fit in a single master curve, showing a tendency of the threshold magnetic field to decrease for the dehydrated samples with respect to the pristine samples. This interesting result points to a positive influence of the SCDP on the actuation behavior of our cross-shaped actuators. It seems that the potential changes on the microstructure of the magnetic hydrogels caused by SCDP facilitated their bending response under a magnetic torque.

## 3. Conclusions

In this study, we have investigated the influence of dehydration on the mechanical properties of alginate hydrogels. We have found that dehydration results in the increase of the robustness of the hydrogel, which in part can be connected to the increase in the concentration of the polymer fibers, as predicted by classical theories of the mechanical properties of networks and gels. However, this increase of robustness is significantly larger for hydrogels subjected to compressive or tensile stress during dehydration than for nondehydrated (control) hydrogels containing a similar polymer concentration. This fact can only be due to changes at the level of microscopic arrangement between polymer fibers, caused by these stress-controlled dehydration processes. Indeed, we have corroborated that reversible rehydration after partial dehydration is impossible for these hydrogels, which may be likely due to the appearance of hydrogen bonds between the -OH groups of the alginate fibers during dehydration. This hypothesis is reinforced by the mechanical anisotropy exhibited by hydrogel samples dehydrated under compressive stress. A microscopic analysis of the fiber network by SEM also seems to provide evidence for the existence of anisotropy, characterized by the appearance of polymer bands observed in certain samples. Additionally, we have reported results of the influence of the stress-controlled dehydration processes on the response under a magnetic field of a cross-shaped magnetic actuator. The actuator consists of a central nonmagnetic hydrogel beam, connected to two lateral magnetic hydrogel beams that bend in response to magnetic fields applied in the perpendicular direction. For all samples, we have observed a threshold value of the magnetic field, which is required to obtain a significant bending of the magnetic hydrogel beams. Interestingly, this threshold value shows a tendency to decrease for samples subjected to dehydration under compression with respect to nondehydrated (control) samples containing similar concentration of magnetic particles. This interesting result points to a positive influence of the changes at the microscopic level caused by the dehydration under compression on the actuation response of magnetic hydrogels. To sum up, our work demonstrates the potential of dehydration under compressive or tensile stress to enhance the properties of hydrogels beyond the limit corresponding to the increase in the concentration of polymers.

## 4. Materials and Methods

### 4.1. Synthesis of Nonmagnetic and Magnetic Hydrogels

In this work, two different sodium alginates (empirical formula ((C_6_H_7_O_6_Na)_n_) were used as a polymer material for the preparation of the hydrogels: low-viscosity sodium alginate (LVSA) of molecular weight 120,000–160,000 g/mol (Sigma Aldrich, St. Louis, MO, USA) and medium-viscosity sodium alginate (MVSA) of molecular weight 10,000–600,000 g/mol (PanReac AppliChem ITW Reagents, Barcelona, Spain). These differences in molecular weight are reflected in the viscosity of the 1 wt.% solutions in the water of both alginates, ranging from 15–25 mPas for Sigma-Aldrich sodium alginate and to 350–550 mPas for sodium alginate from PanReac AppliChem, according to the respective manufacturers, which should be connected with a larger average molecular weight of the MVSA than the LVSA. However, molecular weight distributions are also different, with a broader distribution in the case of MVSA than LVSA, which may also have an effect. Alginate hydrogels are usually prepared by adding a source of divalent cations to a sodium alginate solution [38]. Here, in order to prepare isotropic nonmagnetic and magnetic hydrogels, a two-step protocol reported in a previous work [62] was used with small modifications. Briefly, to synthesize the nonmagnetic hydrogels for the first part of this work, we prepared 12 mL of sodium alginate solutions in distilled water at concentrations of 4.0, 4.5, 5.0, and 5.5 wt.% and then added 36 mg of CaCO_3_ (Sigma Aldrich, St. Louis, MO, USA) and homogenized using a vortex mixer. Afterwards, we added 128.2 mg of D-glucono-δ-lactone (GDL, Sigma Aldrich, St. Louis, MO, USA ) and homogenized the resulting mixture. Then, we poured the mixture into a square container (80.85 mm × 80.85 mm × 2 mm) and left the solution gel undisturbed at room temperature for 2 hours. After this time, we added to the gel 12 mL of a 0.5 M CaCl_2_ (Sigma Aldrich, St. Louis, MO, USA ) solution in distilled water, and the sample was left overnight at room temperature for complete gelation. We prepared two types of these hydrogels, one containing only LVSA (AH100-0) and another containing a mixture of 50% LVSA and 50% MVSA (AH50-50) (see Table 3).

In the case of hydrogels used for the actuator, we had to prepare two different parts: a magnetic active one, which responded to the magnetic field applied, and a nonmagnetic passive one that remained unaltered. For the magnetic part of the actuator, the previous protocol was slightly modified to introduce magnetic microparticles within the composition, as described in what follows. To 10 mL of a solution of MVSA in distilled water at a concentration of 1 wt.%, 15 mg of CaCO_3_ were added, followed by homogenization, and subsequent addition of 53.4 mg of GDL and further homogenization. Then, proper amounts of silica-coated iron powder (this powder consisted of spherical micronsized particles of diameter 1.4 ± 0.6 µm Fe-CC, BASF, Ludwigshafen, Germany) were added to obtain the desired concentration of magnetic particles in the final hydrogels (see Table 3) and homogenized by vortex mixing and 10 min of ultrasonic bath. Then, the mixture was poured in a cross-shaped mold and waited for two hours at room temperature. Afterwards, 10 mL of a 45 mM aqueous solution of CaCl_2_ was added and the sample was left overnight at room temperature for complete gelation. The nonmagnetic part of the magnetic actuators was prepared in the same way, but without introducing magnetic particles to the formulation. Note that in this case, we only used MVSA at a concentration of 1 wt.% in order to obtain an appropriate stiffness of the polymer matrix to maximize the magnetic response of the hydrogels. With this alginate type and concentration, we managed to obtain a flexible actuator, which maintained an adequate consistence for manipulation.

### 4.2. Stress-Controlled Dehydration Processes (SCDP)

We used two different stress-controlled dehydration processes based on the methods described before [36,37], differentiated by the type of stress applied during dehydration: tensile or compressive stress (Figure S5a). The hydrogels used in these processes were C4.0AH100-0 and C4.0AH50-50, where the total alginate concentration before the application of the SCDP was 4 wt.%. To dehydrate these hydrogels under a tensile stress, a home-made device, which fixed the two extremities of the sample and stretched it was used (Figure S5b,c). First, the hydrogels were cut in a rectangular shape (25 mm × 50 mm × 2 mm). Then, two of these gels were simultaneously fixed and slightly stretched in the device (Figure S5c). Afterwards, the system was placed into a desiccator at room temperature, and a partial vacuum was made. We left the samples dehydrating for 8, 14, and 24 h to obtain different water losses. During the process, the relative humidity inside the desiccator was regulated by a 25 wt.% sodium hydroxide (NaOH, Sigma Aldrich, St. Louis, MO, USA) solution in distilled water, which maintained a relative humidity of 60% at 25 °C [67]. Note that, due to the long times of dehydration, the control of temperature and relative humidity was crucial to obtain different reproducible degrees of water losses. During the described process, the hydrogels shrank because of the evaporated water, generating a rising tensile stress in the longitudinal direction of the sample since the two extremities of the hydrogel remained fixed. The increasing tensile stress during the dehydration was the reason behind the changes studied here, and not the initial negligible stress applied to the hydrogels with the home-made device.

For the dehydration of the nonmagnetic hydrogels under compressive stress, we placed square-like (28 mm × 28 mm × 2 mm) hydrogel samples between two glass plates surrounded by blotting paper and positioned a mass of 6.5 kg on the top of the upper plate (Figure S5d). Then, we let them dry at room temperature for 10, 20, and 30 minutes, obtaining different reproducible degrees of dehydration. In the case of hydrogels constituting the magnetic actuator, because of the much softer consistency, we placed the hydrogels between two pieces of filter paper and compressed them with a much smaller mass (96 g) and time (90 s), repeating the process twice for some specific samples. In the case of compression, it was not necessary to control the temperature and relative humidity due to the experimental setup and the rapidity of the process. In addition, note that since our focus was on the final concentration of polymer fibers after dehydration and because kinetics of different hydrogels and different process (compression and traction) were different, we used rather dissimilar times of dehydration in each case.

### 4.3. Mechanical Characterization of the Alginate Hydrogels

For the characterization of the mechanical properties, we subjected the hydrogels separately to uniaxial tensile stress and oscillatory shear stress. The tensile tests were carried out using a hybrid rheometer (Discovery HR-1, TA Instruments, New Castle, DE, USA) at room temperature. For this, the samples were cut into the shape of a dog bone and clamped between the two clamps of the rheometer. The clamps had grooved surface that prevented the sample from slipping. During the measurement, the lower clamp remained static while the upper clamp moved. In all cases, we applied a stretching preload of 0.3 N normal axial force to set a reproducible starting condition. The hydrogels were then stretched at a constant rate of 50.0 µm/s until breakage. Then, from the obtained engineering stress vs. strain curves, the Young’s modulus (*E*), the strain at break (*ε_b_*), and the stress at the break (*σ_b_*) of the samples were calculated. Young’s modulus was calculated as the slope of the linear regression of strain (*ε*) versus tensile stress (*σ*) (Equation (4)) [68]:

(4) $$E=\frac{F/S}{\left(l-l_{0}\right)/l_{0}}=\frac{\sigma }{\varepsilon },$$

where *l* is the length of the sample during the test, *l*_0_ the initial length of the sample, *F* is the force applied, and *S* is the cross-section of the sample. Finally, the strain and stress at break were obtained from the last point of the curves before the sample breakage.

The characterization of the viscoelastic behavior of the alginate hydrogels under oscillatory shear was carried out using a rotational rheometer (Physica MCR 300, Anton Paar, Graz, Austria) with a plate–plate geometry of 20 mm of diameter at a constant temperature of 25.0 ± 0.1 °C. For this purpose, the hydrogels were placed on the lower plate of the measuring system and the upper plate was descended until contact with the top surface of the sample. To ensure reproducibility, the hydrogels were subjected to a constant normal force of 0.1 N for 100 s before the rheological measurement. This normal force was also maintained during the entire duration of the experiment in order to guarantee proper contact between the hydrogel and the geometry of the rheometer during shear oscillations. We analyzed the rheological behavior of the samples by amplitude sweeps and frequency sweeps. In amplitude sweeps, we subjected the samples to the oscillatory shear strain of increasing strain amplitude (logarithmic ramp from γ_i_ = 0.001% to γ_f_ = 100%) and fixed frequency ν_0_ = 1 Hz. From these measurements, we obtained the values of the storage modulus *G*’ and loss modulus *G*″ as a function of the shear strain γ. In frequency sweeps, we subjected the samples to the oscillatory shear of constant strain amplitude (γ_0_ = 0.01%) and increasing frequency (logarithmic ramp from ν_i_ = 0.1 Hz to ν_f_ = 20 Hz). From these measurements, we obtained *G*’ and *G*″ as a function of frequency. Each sweep (amplitude or frequency) contained a total of 30 steps for which the same shear amplitude and frequency were maintained for 10 s.

### 4.4. Scanning Electron Microscopy Imaging of the Hydrogel Microstructure

The microscopic structure of the alginate hydrogels was characterized by scanning electron microscopy (SEM) using a HITACHI S-510 microscope.

### 4.5. Analysis of the Actuation Behavior

For the analysis of the actuation behavior of the magnetic hydrogels, we constructed a planar cross-shaped actuator consisting of two magnetic hydrogel beams (active parts) connected to a central nonmagnetic hydrogel beam (passive part). To do this, we used a cross-shaped mold and poured the precursor mixtures of the nonmagnetic hydrogel and the magnetic hydrogel into its different zones, separated by a ring (Figure S7), then removing the ring and proceeding to cross-link them in the same way described above in Section 4.1 After the gelation was completed, the actuator was placed on an aluminum support at medium height inside an electric coil connected to a DC power supply, which was used for the application of the magnetic field. We then investigated the bending of the magnetic hydrogel beams for stepwise increasing values of the applied magnetic field up to a maximum field strength of 60 kA/m, afterwards decreasing the field to zero to investigate potential hysteresis of the actuation response. For each value of the applied field, we waited for 30 s before measuring the bending angle with respect to the off-state. Note that because the lack of continuity of the adhesion forces between the actuator and the support, and since we were interested only in the balance between magnetic, elastic, and gravitational forces, for increasing magnetic field starting at zero field, we manually detached the magnetic hydrogel beams from the support surface and waited until equilibrium. If equilibrium was reached at an angle of zero, we increased the field and proceeded in the same way. Once the equilibrium was reached at an angle larger than zero, we increased the field stepwise, and no manual detachment was further needed. Note that the bending angles were calculated from the values of the height of the free end of the magnetic beams with respect to the off-state position, which were measured directly (Figure S2).

### 4.6. Statistics

For each set of experimental conditions, at least three different samples were measured, and the corresponding mean value and standard deviation are provided. When differences between values corresponding to different samples and/or experimental conditions were not clear, we applied t-tests with a significance level of 0.05 to discern the statistical significance of the differences. For more details related to these tests, see Tables S1–S3.

## Figures and Tables

**Table 1:** Hydrogels mean alginate concentration (c) and water lost (wl) after the two SCDP carried out during different times. Alginate concentration before the SCDP was 4 wt.% (C4.0AH100-0 and C4.0AH50-50) in all cases, as been mentioned before.

Stress Applied	Time	AH100-0		AH50-50	
		c (wt.%)	wl (wt.%)	c (wt.%)	wl (wt.%)
Compressive	10 min	4.72 ± 0.21	16 ± 4	4.41 ± 0.04	9.8 ± 0.9
	20 min	5.00 ± 0.16	21 ± 3	4.61 ± 0.07	13.8 ± 1.4
	30 min	5.3 ± 0.3	26 ± 5	4.79 ± 0.07	17.1 ± 1.3
Tensile	8 h	4.67 ± 0.07	15.0 ± 1.4	4.87 ± 0.11	19 ± 2
	14 h	4.86 ± 0.12	18 ± 2	5.34 ± 0.10	26.1 ± 1.6
	24 h	5.48 ± 0.21	28 ± 3	6.2 ± 0.3	37 ± 3

**Table 2:** Magnetic particle concentration (Fe-CC) and alginate concentration (c) of the different hydrogels after the first and second dehydration (FD and SD, respectively).

	Fe-CC (% *v*/*v*)		c (wt.%)	
	FD	SD	FD	SD
M5AH	7.3 ± 1.4	8.0 ± 1.2	1.5 ± 0.3	1.7 ± 0.3
M7.5AH	9.7 ± 1.6	12.2 ± 2.5	1.33 ± 0.23	1.7 ± 0.4
M10AH	14.7 ± 1.2	18.4 ± 1.8	1.56 ± 0.15	2.03 ± 0.23
M20AH	26 ± 5	36 ± 6	1.4 ± 0.4	1.9 ± 0.6

**Table 3:** Concentrations of LVSA, MVSA and Fe-CC in all the synthesized nonmagnetic and magnetic hydrogels. CXAH100-0 and CXAH50-50 are the control samples where X is the total alginate concentration. MYAH are the hydrogels for the actuator where Y is the concentration of magnetic particles.

Sample	wt.% LVSA	wt.% MVSA	% *v*/*v* Fe-CC
C4.0AH100-0	4.00	0	0
C4.0AH50-50	2.00	2.00	0
C4.5AH100-0	4.50	0	0
C4.5AH50-50	2.25	2.25	0
C5.0AH100-0	5.00	0	0
C5.0AH50-50	2.50	2.50	0
C5.5AH100-0	5.50	0	0
C5.5AH50-50	2.75	2.75	0
M0AH	0	1.00	0
M5AH	0	1.00	5.0
M7.5AH	0	1.00	7.5
M10AH	0	1.00	10.0
M20AH	0	1.00	20.0

## Data Availability

The datasets supporting this article is available using Figshare: https://doi.org/10.6084/m9.figshare.21802479 (accessed on 26 December 2022).

## Supplementary Materials

The following supporting information can be downloaded at: https://www.mdpi.com/article/10.3390/gels9010039/s1, Figure S1: Macroscopic appearance of C4.0AH100-0 (bottom) and C4.0AH50-50 (top) immediately after their preparation (a) and after the SCDPs (b) (see Section 4). The square samples are for the SCDP under compressive stress and the rectangular ones for the SCDP under tensile stress. Scale bars correspond to 15 mm.; Figure S2: Deformation at break and tensile stress at break of the hydrogels measured under tensile stress. In this figure are represented the data of the control samples, the samples dehydrated under compressive and tensile stress. The bar with solid colors are the values for AH100-0 and the striped ones for AH50-50. (a) Maximum deformation at break. (b) Tensile stress at break. Note that the same scale is used in the axis.; Figure S3: Loss modulus of the hydrogels measured under oscillatory shear stress. In this figure is represented the data of the control samples, the samples dehydrated under compressive and tensile stress. The bar with solid colors are the values for AH100-0 and the striped ones for AH50-50. Note that the same scale is used in the axes.; Figure S4: Oscillatory frequency sweeps of the dehydrated alginate hydrogels. (a) AH100-0 and (b) AH50-50. Note that same scales are used in both axes.; Figure S5: (a) Sketch with the directions of the stresses applied to the alginate hydrogels during the SCDPs. (b,c) sketch and picture, respectively, of the device used in the dehydration process under tensile stress. (d) Sketch of the dehydration process under compressive stress.; Figure S6: SEM images of the alginate hydrogels studied. (a,b) polymeric structure of AH100-0 and AH50-50 after the SCDP under compressive stress (side view), respectively. (c,d) polymeric structure of AH100-0 and AH50-50 after the SCDP under compressive stress (top view), respectively. The compressive stress in all cases was applied during 30 minutes. Scale bars correspond to 1 µm.; Figure S7: (a) Sketch of the mold and the soft magnetic actuator. (b) Approximation used for the calculation of the angle of the magnetic beam of the actuator.; Figure S8: Angle vs. Magnetic field intensity for all the magnetic particles concentration and dehydration of the actuator. FD: First dehydration; SD: Second Dehydration; I.: Increasing; D.: Decreasing. Note that same scales are used in both axes.; Table S1: Two samples *t*-test with a significance level of 0.05 and null hypothesis mean1-mean2=0 for the magnitudes from the uniaxial tensile tests measured parallel to the direction of the tensile stress applied during the SCDPs. Column a: equal variance is assumed. Column b: equal variance is not assumed.; Table S2: Two samples *t*-test with a significance level of 0.05 and null hypothesis mean1-mean2=0 for the magnitudes from the oscillatory shear tests. Column a: equal variance is assumed. Column b: equal variance is not assumed.; Table S3: Two samples *t*-test with a significance level of 0.05 and null hypothesis mean1-mean2=0 for the magnitudes from the uniaxial tensile tests measured perpendicularly to the direction of the tensile stress applied during the SCDPs. Column a: equal variance is assumed. Column b: equal variance is not assumed.
