# Supplementary material for: Alginate Hydrogels Reinforced by Dehydration under Stress—Application to a Soft Magnetic Actuator
Source: Gels. 2023 Jan 3;9(1):39. doi: 10.3390/gels9010039 (PMC9858607; doi:10.3390/gels9010039)
Supplement: Supplementary file 1 [file gels-09-00039-s001.zip › Supplementary_information.pdf]

SUPPLEMENTARY INFORMATION

Alginate hydrogels reinforced by dehydration under stress. Application to a soft magnetic actuator.

Alberto Leon-Cecilla, Francisco J. Vazquez-Perez, Cristina Gila-Vilchez,
Luis Álvarez de Cienfuegos and Modesto T. Lopez-Lopez

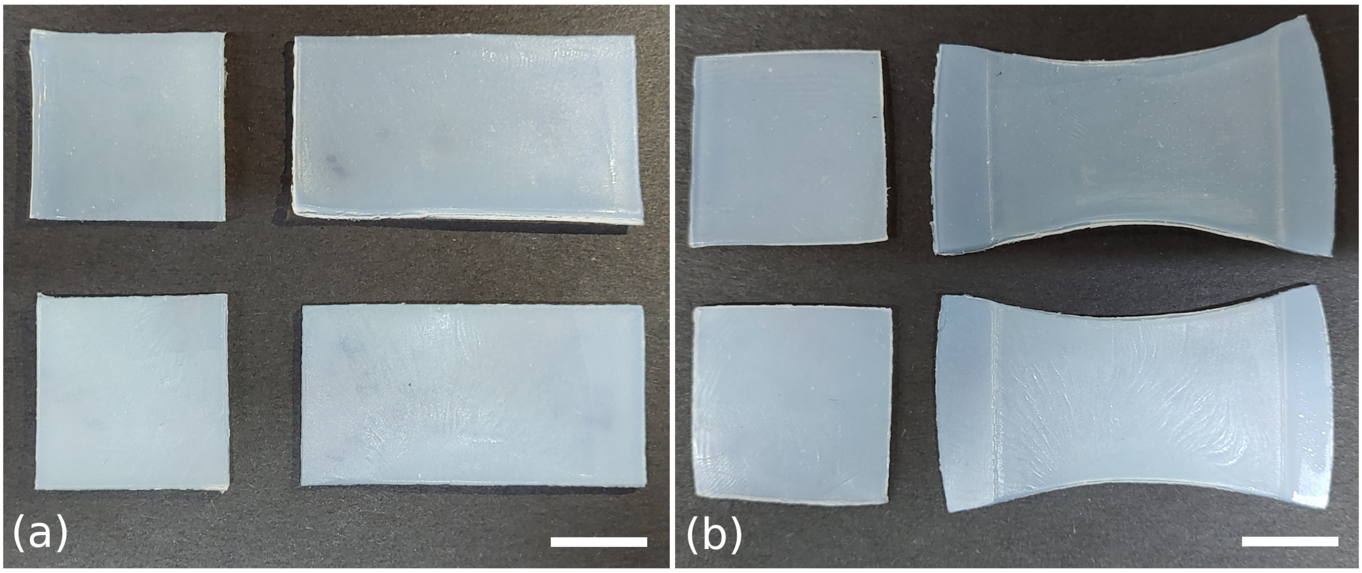

Figure S1: Macroscopic appearance of C4.0AH100-0 (bottom) and C4.0AH50-50 (top) immediately after their preparation (a) and after the SCDPs (b) (see Section 4). The square samples are for the SCDP under compressive stress and the rectangular ones for the SCDP under tensile stress. Scale bars correspond to 15 mm.

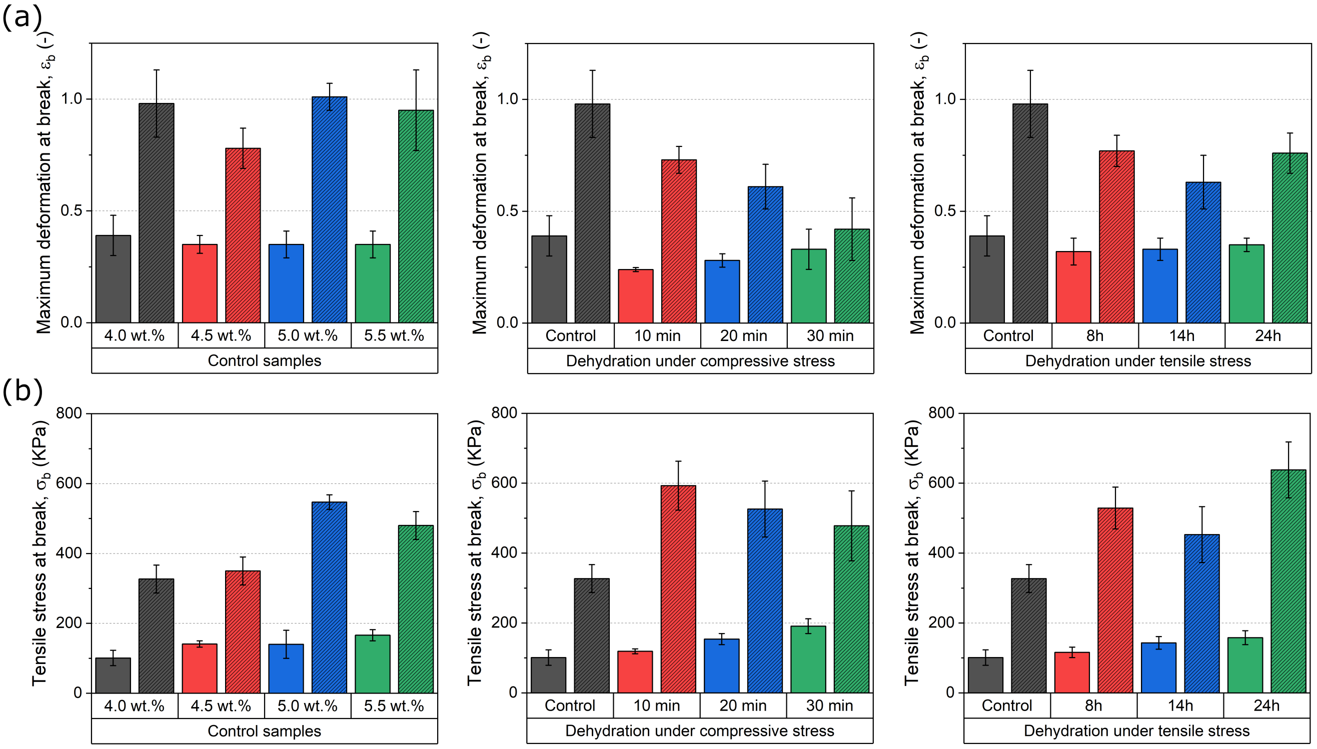

Figure S2: Deformation at break and tensile stress at break of the hydrogels measured under tensile stress. In this figure are represented the data of the control samples, the samples dehydrated under compressive and tensile stress. The bar with solid colors are the values for AH100-0 and the striped ones for AH50-50. (a) Maximum deformation at break. (b) Tensile stress at break. Note that the same scale is used in the axis.

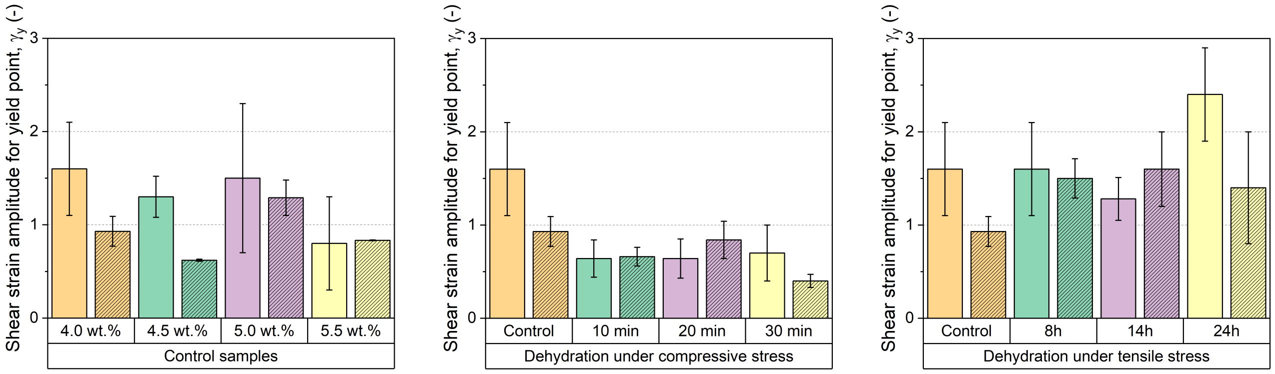

Figure S3: Loss modulus of the hydrogels measured under oscillatory shear stress. In this figure is represented the data of the control samples, the samples dehydrated under compressive and tensile stress. The bar with solid colors are the values for AH100-0 and the striped ones for AH50-50. Note that the same scale is used in the axes.

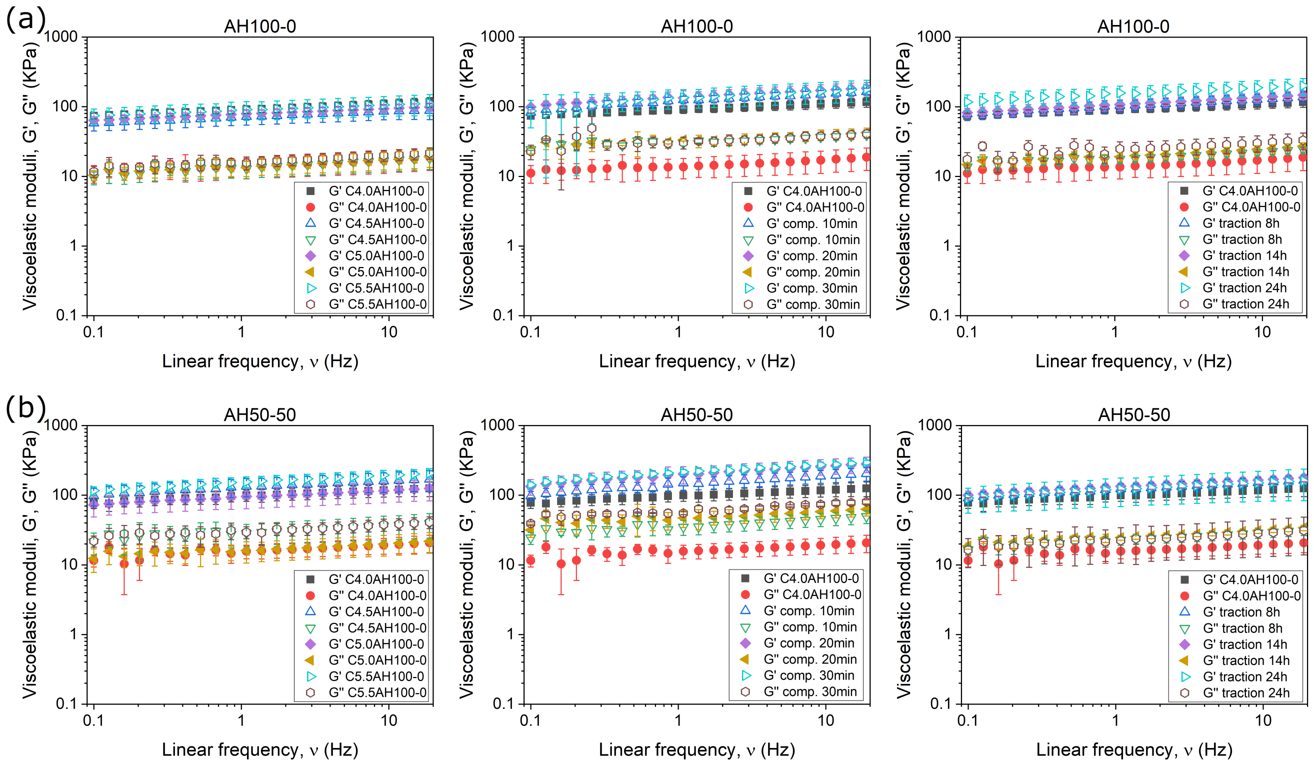

Figure S4: Oscillatory frequency sweeps of the dehydrated alginate hydrogels. (a) AH100-0 and (b) AH50-50. Note that same scales are used in both axes.

Table S1: Two samples t-test with a significance level of 0.05 and null hypothesis mean1-mean2=0 for the magnitudes from the uniaxial tensile tests measured parallel to the direction of the tensile stress applied during the SCDPs. Column a: equal variance is assumed. Column b: equal variance is not assumed.

Magnitude	Samples	a	b
Young's modulus	AH100-0 control 4.5 wt.% & AH100-0 control 5.5 wt.%	✓	✓
	AH100-0 control 5.0 wt.% & AH100-0 control 5.5 wt.%	✓	✓
	AH100-0 traction 8h & AH100-0 traction 14h	✓	✓
	AH100-0 compression 20 min & AH100-0 compression 20 min	X	X
	AH50-50 traction 14h & AH50-50 traction 24h	X	X
	AH50-50 compression 30 min & AH50-50 traction 24h	X	X
Maximum deformation at break	AH100-0 control 4.0 wt.% & AH100-0 compression 20 min	X	X
	AH100-0 compression 10 min & AH100-0 compression 20 min	✓	X
	AH100-0 compression 10 min & AH100-0 compression 30 min	X	X
	AH50-50 control 4.0 wt.% & AH50-50 traction 8h	✓	✓
	AH50-50 control 4.0 wt.% & AH50-50 traction 24h	✓	✓
Tensile stress at break	AH100-0 control 4.5 wt.% & AH100-0 control 5.5 wt.%	✓	✓
	AH100-0 compression 20 min & AH100-0 compression 30 min	✓	✓
	AH100-0 traction 8h & AH100-0 traction 14h	X	X
	AH100-0 compression 30 min & AH100-0 traction 24h	✓	✓
	AH50-50 control 4.0 wt.% & AH50-50 traction 14h	✓	✓
	AH50-50 traction 8h & AH50-50 traction 24h	✓	X
	AH50-50 compression 30 min & AH50-50 traction 24h	✓	✓

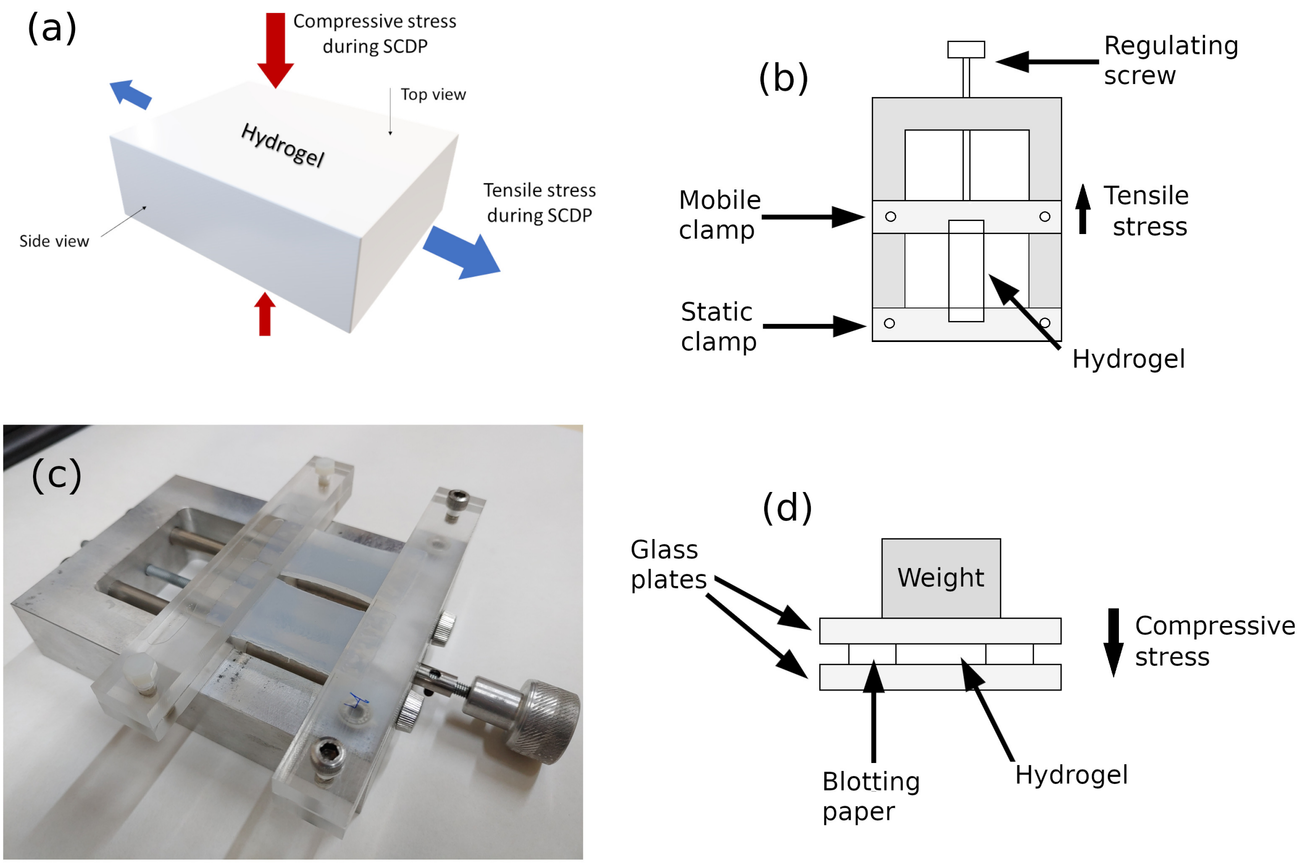

Figure S5: (a) Sketch with the directions of the stresses applied to the alginate hydrogels during the SCDPs. (b) and (c) sketch and picture, respectively, of the device used in the dehydration process under tensile stress. (d) Sketch of the dehydration process under compressive stress.

Table S2: Two samples t-test with a significance level of 0.05 and null hypothesis mean1-mean2=0 for the magnitudes from the oscillatory shear tests. Column a: equal variance is assumed. Column b: equal variance is not assumed.

Magnitude	Samples	a	b
Storage modulus	AH100-0 control 4 wt.% & AH100-0 compression 30 min	X	X
	AH100-0 control 5.5 wt.% & AH100-0 compression 30 min	X	X
	AH100-0 control 4 wt.% & AH100-0 traction 14h	X	X
	AH100-0 control 5 wt.% & AH100-0 traction 14h	✓	X
	AH100-0 compression 10 min & AH100-0 traction 8h	X	X
	AH100-0 compression 20 min & AH100-0 traction 14h	X	X
Loss modulus	AH100-0 control 4 wt.% & AH100-0 traction 24h	X	X
	AH100-0 control 5 wt.% & AH100-0 traction 14h	X	X
	AH100-0 control 5.5 wt.% & AH100-0 traction 24h	X	X
	AH100-0 traction 8h & AH50-50 traction 24h	X	X
	AH50-50 compression 10 min & AH50-50 traction 8h	X	X

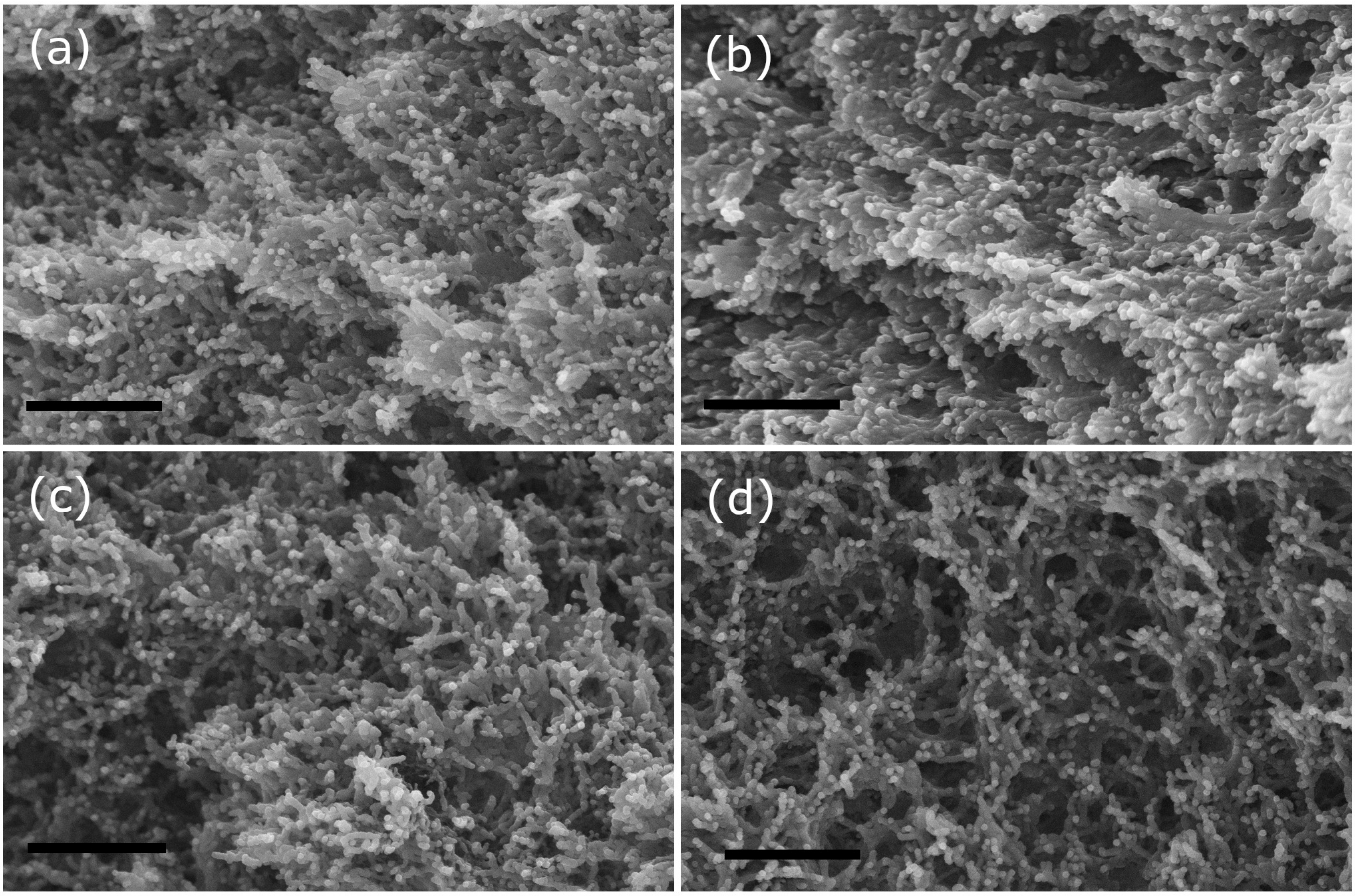

Figure S6: SEM images of the alginate hydrogels studied. (a) and (b) polymeric structure of AH100-0 and AH50-50 after the SCDP under compressive stress (side view), respectively. (c) and (d) polymeric structure of AH100-0 and AH50-50 after the SCDP under compressive stress (top view), respectively. The compressive stress in all cases was applied during 30 minutes. Scale bars correspond to 1 μm.

Table S3: Two samples t-test with a significance level of 0.05 and null hypothesis mean1-mean2=0 for the magnitudes from the uniaxial tensile tests measured perpendicularly to the direction of the tensile stress applied during the SCDPs. Column a: equal variance is assumed. Column b: equal variance is not assumed.

Magnitude	Samples	a	b
Young's modulus	AH100-0 control 4 wt.% & AH100-0 traction 24h perpendicular	✓	✓
	AH100-0 control 4.0 wt.% & AH50-50 control 4.0 wt.%	✓	✓
	AH100-0 traction 24h perpendicular & AH50-50 traction 24h perpendicular	X	✓
Tensile stress at break	AH100-0 control 4.0 wt.% & AH100-0 traction 24h perpendicular	X	X
	AH50-50 traction 24h & AH50-50 traction 24h perpendicular	✓	✓
Deformation at break	AH100-0 traction 24h & AH100-0 traction 24h perpendicular	X	X
	AH50-50 control 4.0 wt.% & AH50-50 traction 24h	✓	✓

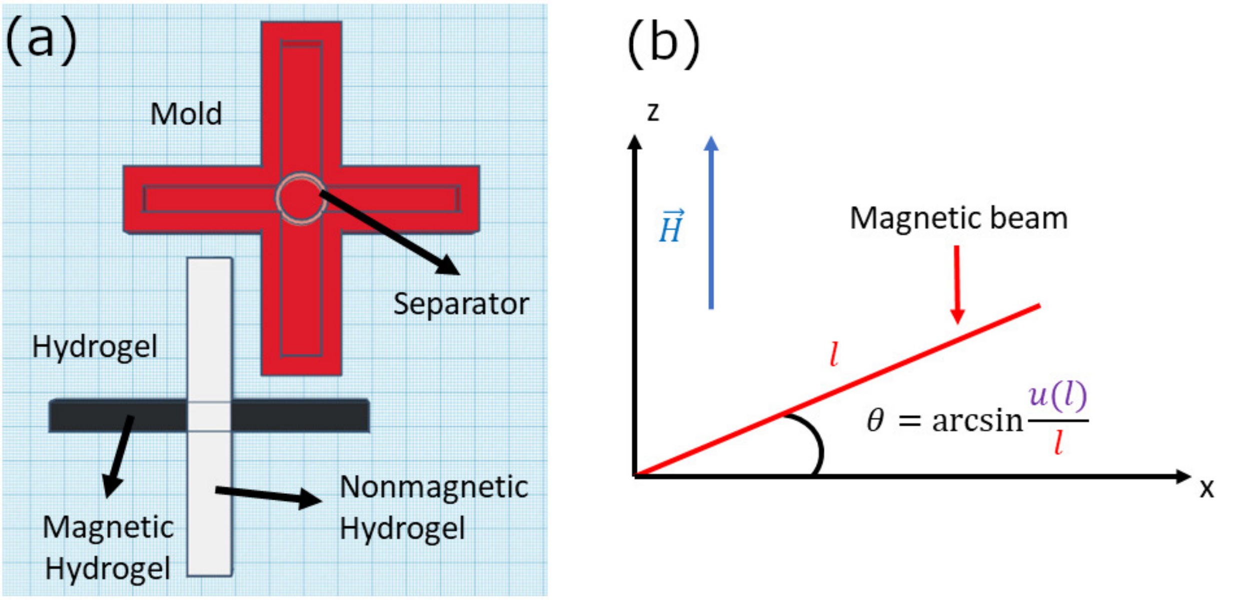

Figure S7: (a) Sketch of the mold and the soft magnetic actuator. (b) Approximation used for the calculation of the angle of the magnetic beam of the actuator.

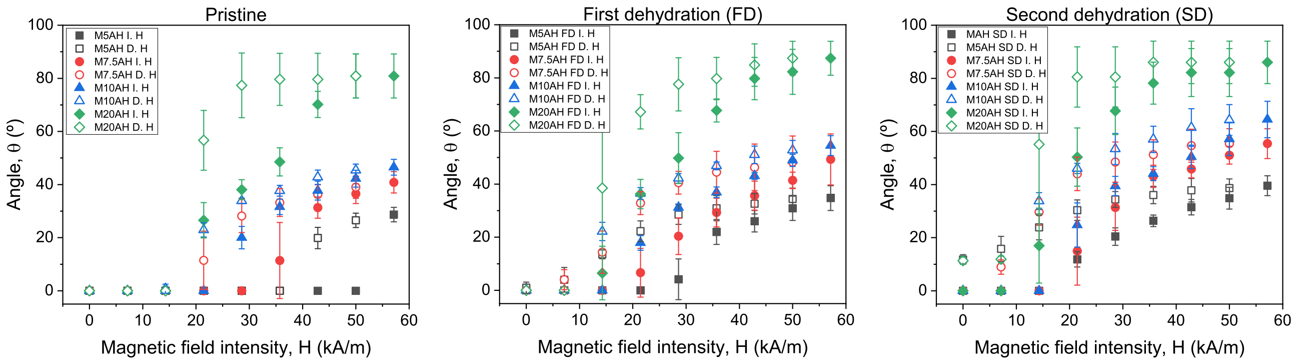

Figure S8: Angle vs. Magnetic field intensity for all the magnetic particles concentration and dehydration of the actuator. FD: First dehydration; SD: Second Dehydration; I.: Increasing; D.: Decreasing. Note that same scales are used in both axes.
