# Supplementary figures and images for: Alginate Hydrogels Reinforced by Dehydration under Stress—Application to a Soft Magnetic Actuator
Source: Gels. 2023 Jan 3;9(1):39. doi: 10.3390/gels9010039 (PMC9858607; doi:10.3390/gels9010039)

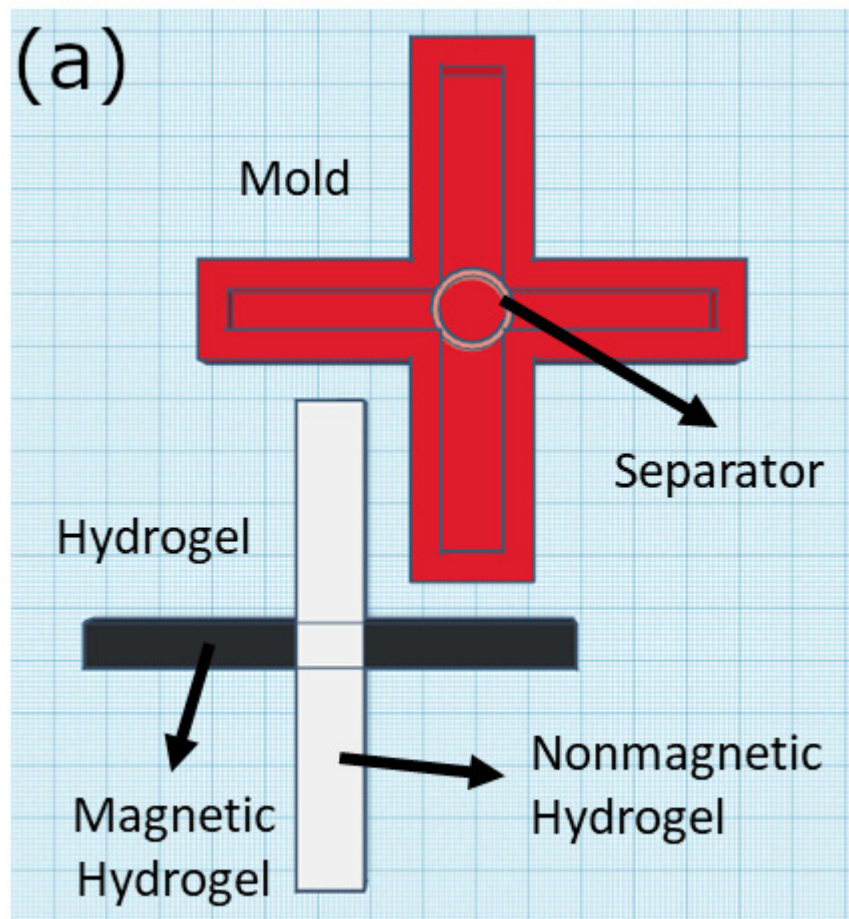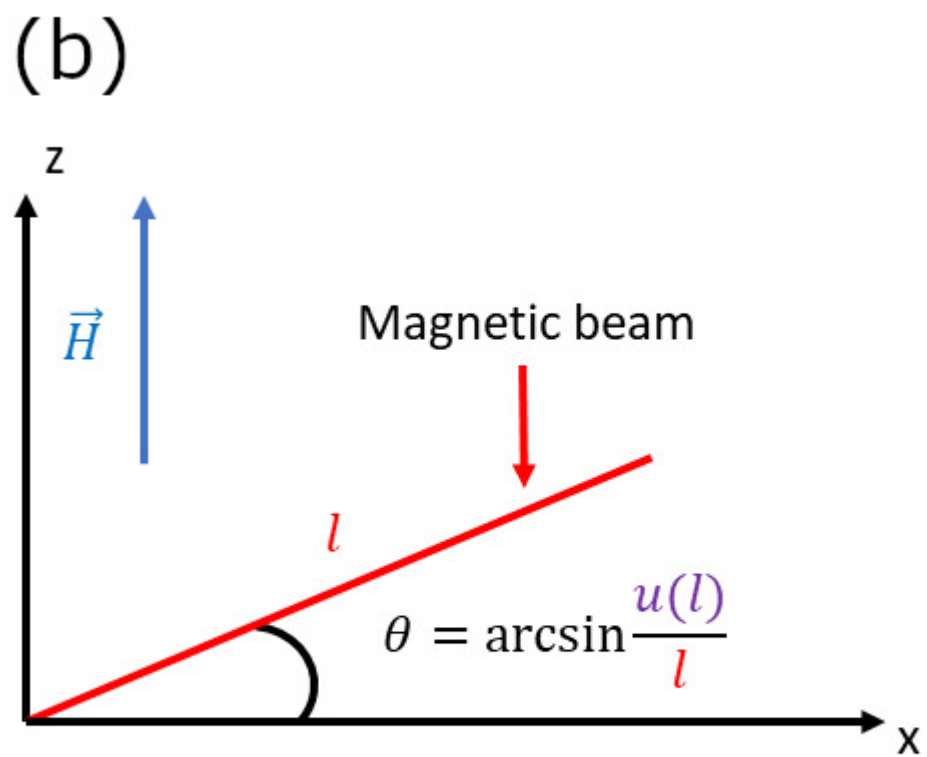

Supplement: Supplementary file 1 [file gels-09-00039-s001.zip › Figures_supplementary/Actuator_supp-eps-converted-to.pdf]

Pristine

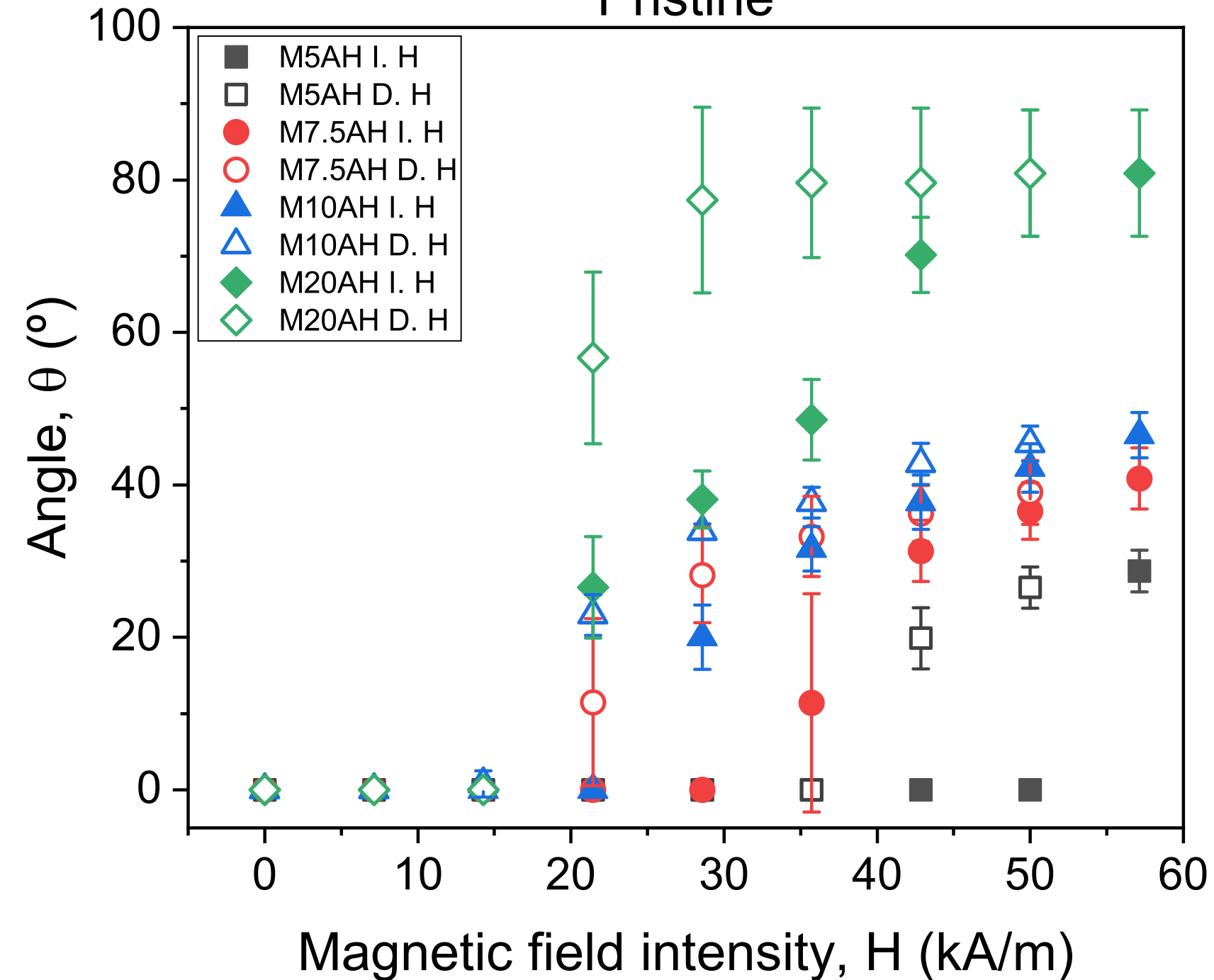

First dehydration (FD)

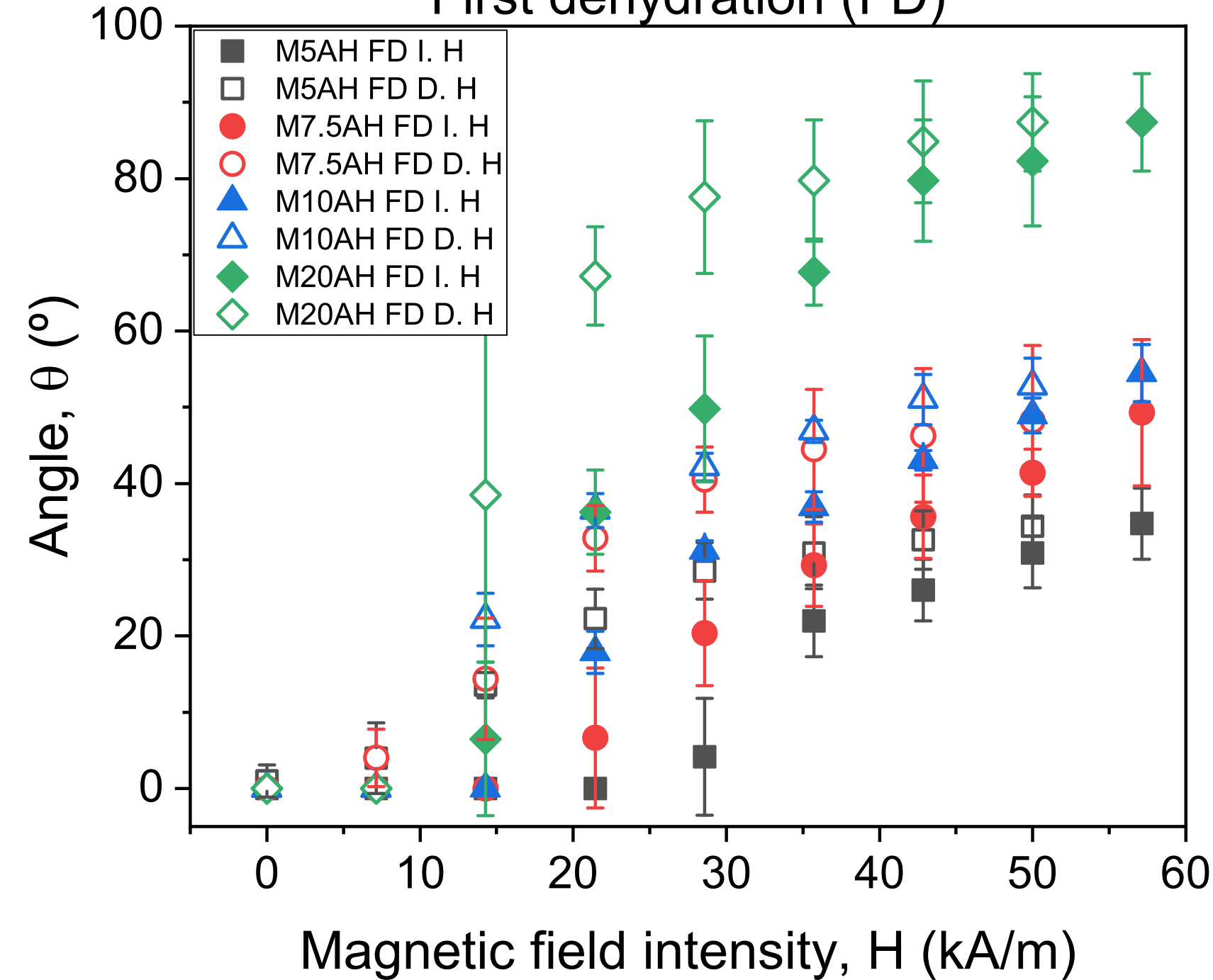

Second dehydration (SD)

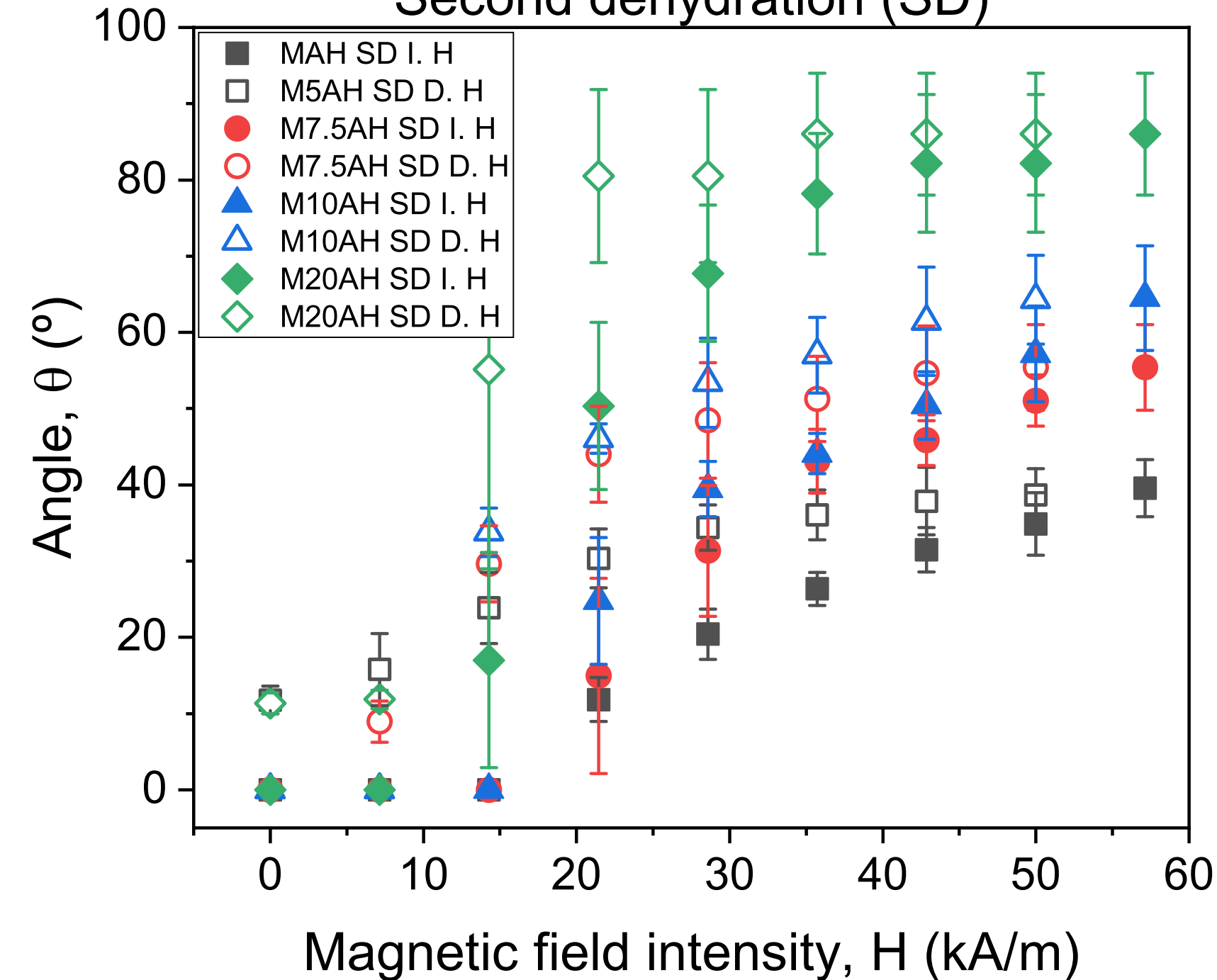

Supplement: Supplementary file 1 [file gels-09-00039-s001.zip › Figures_supplementary/Angle_field-eps-converted-to.pdf]

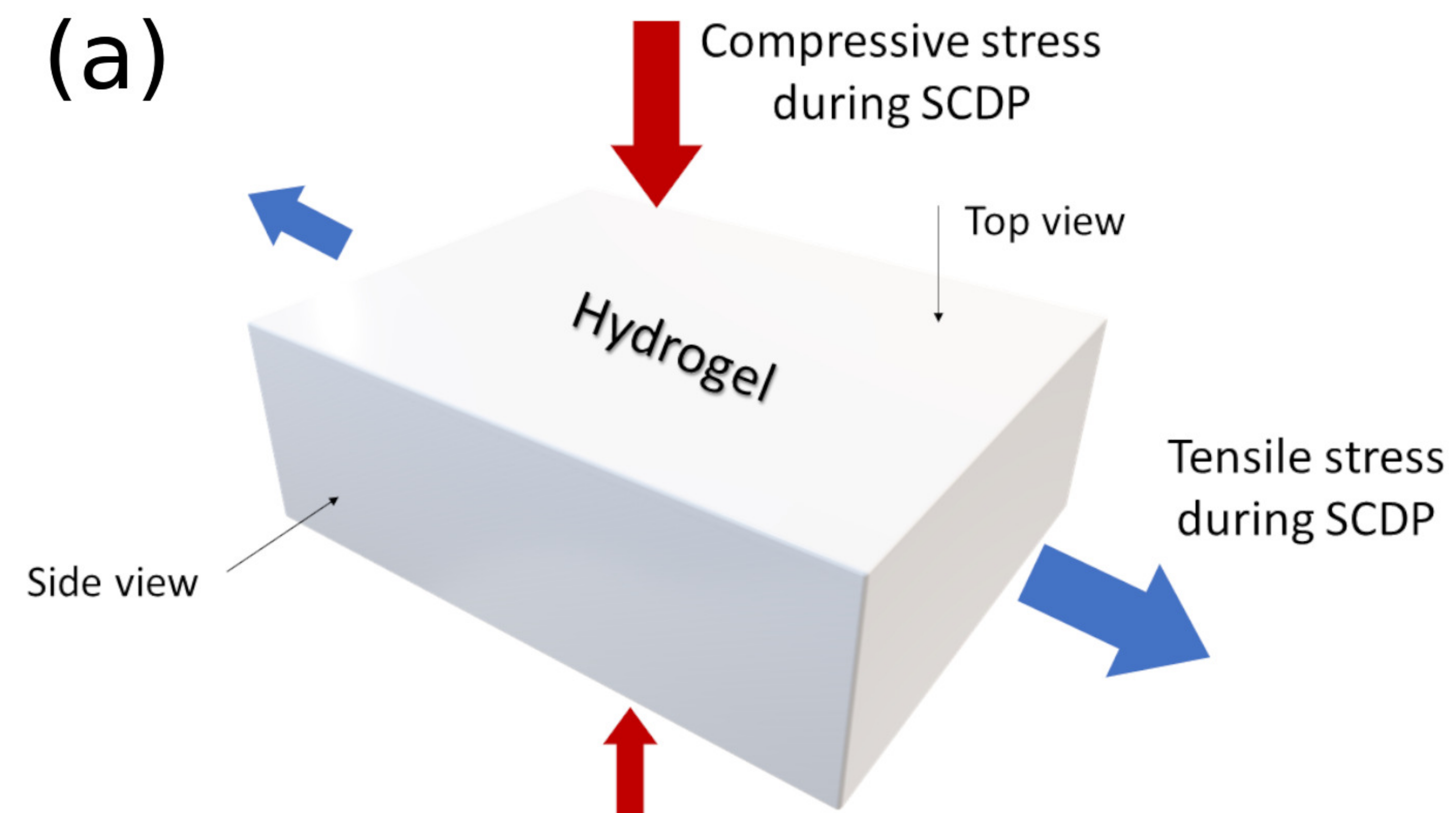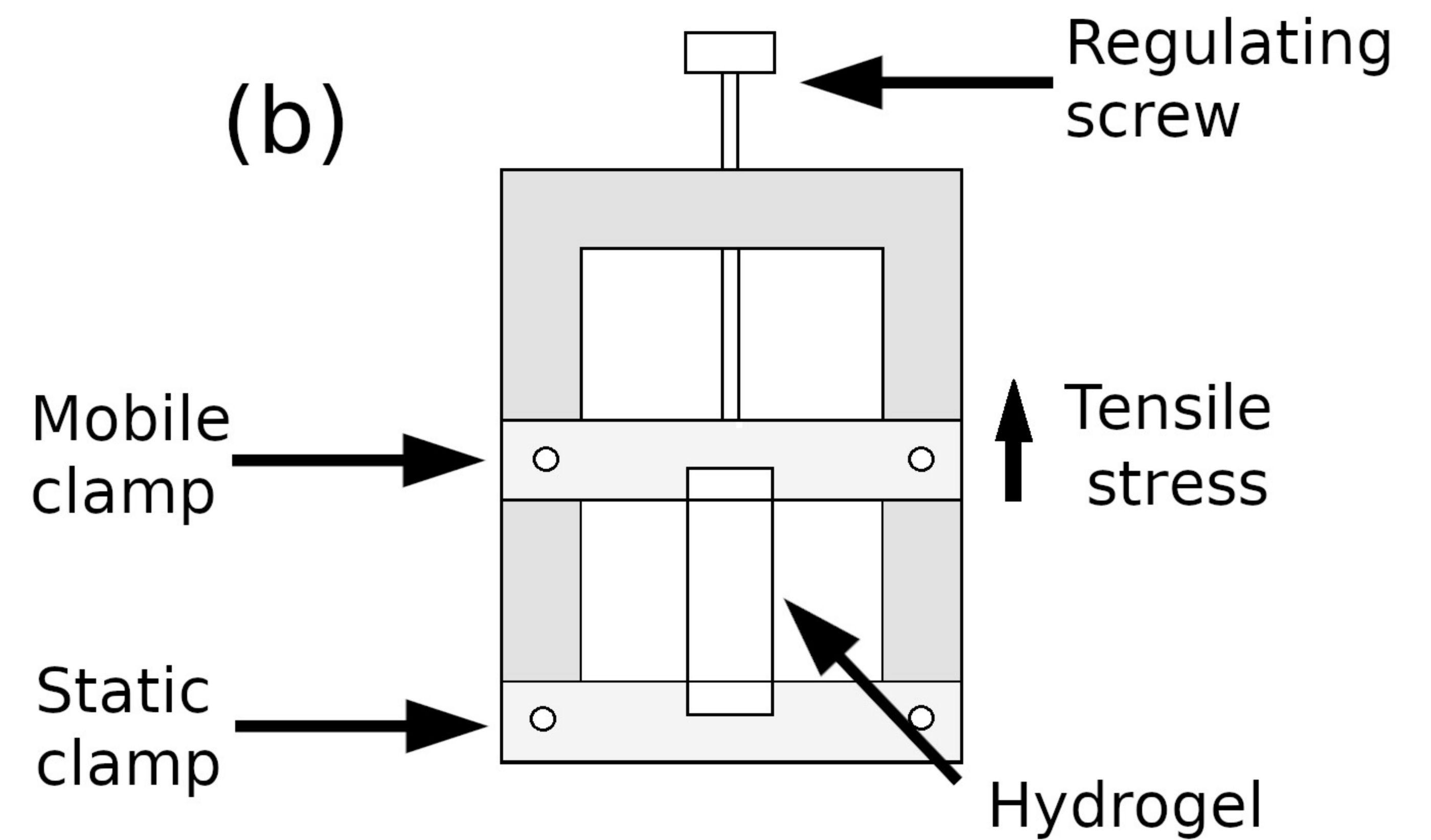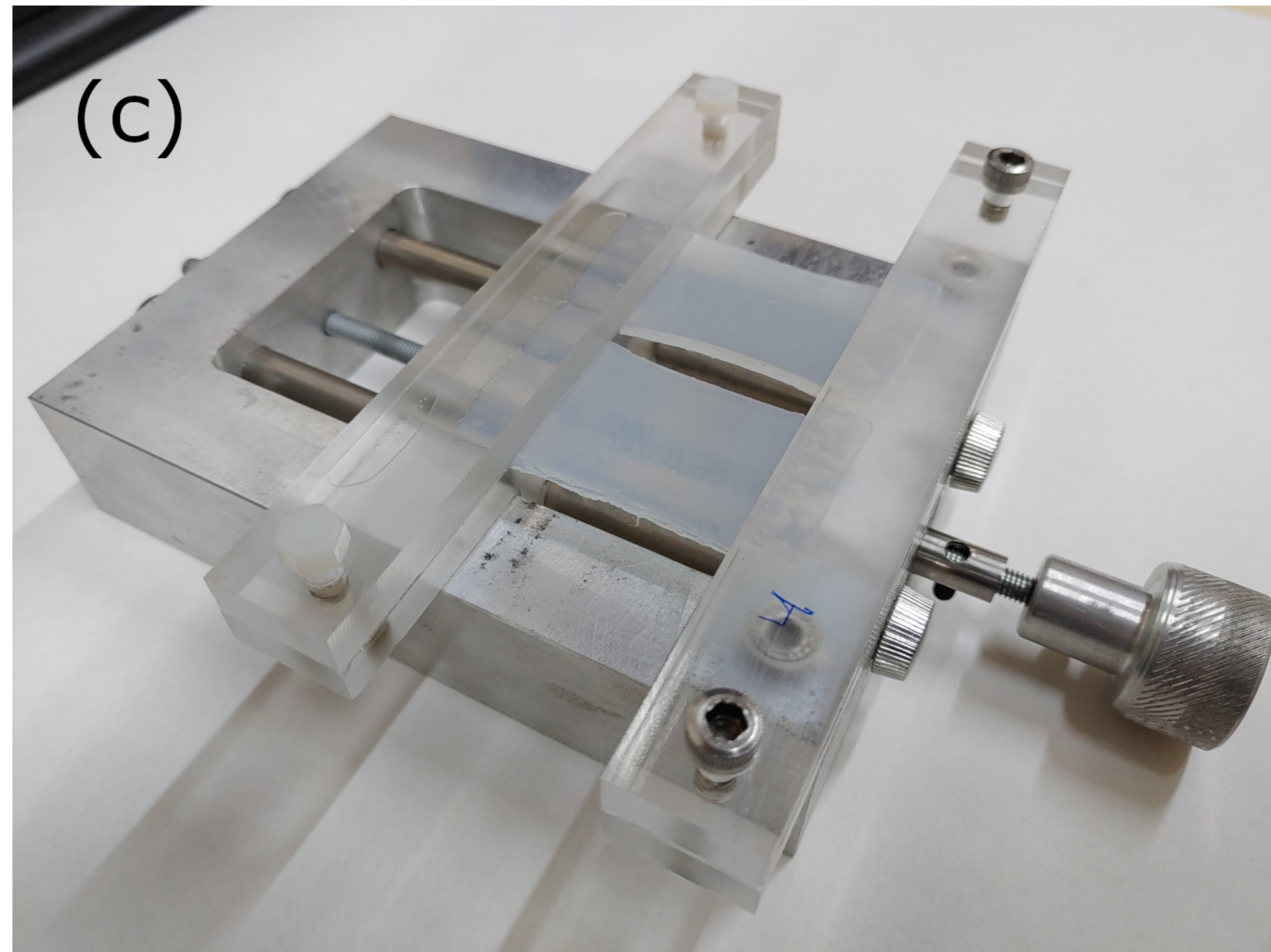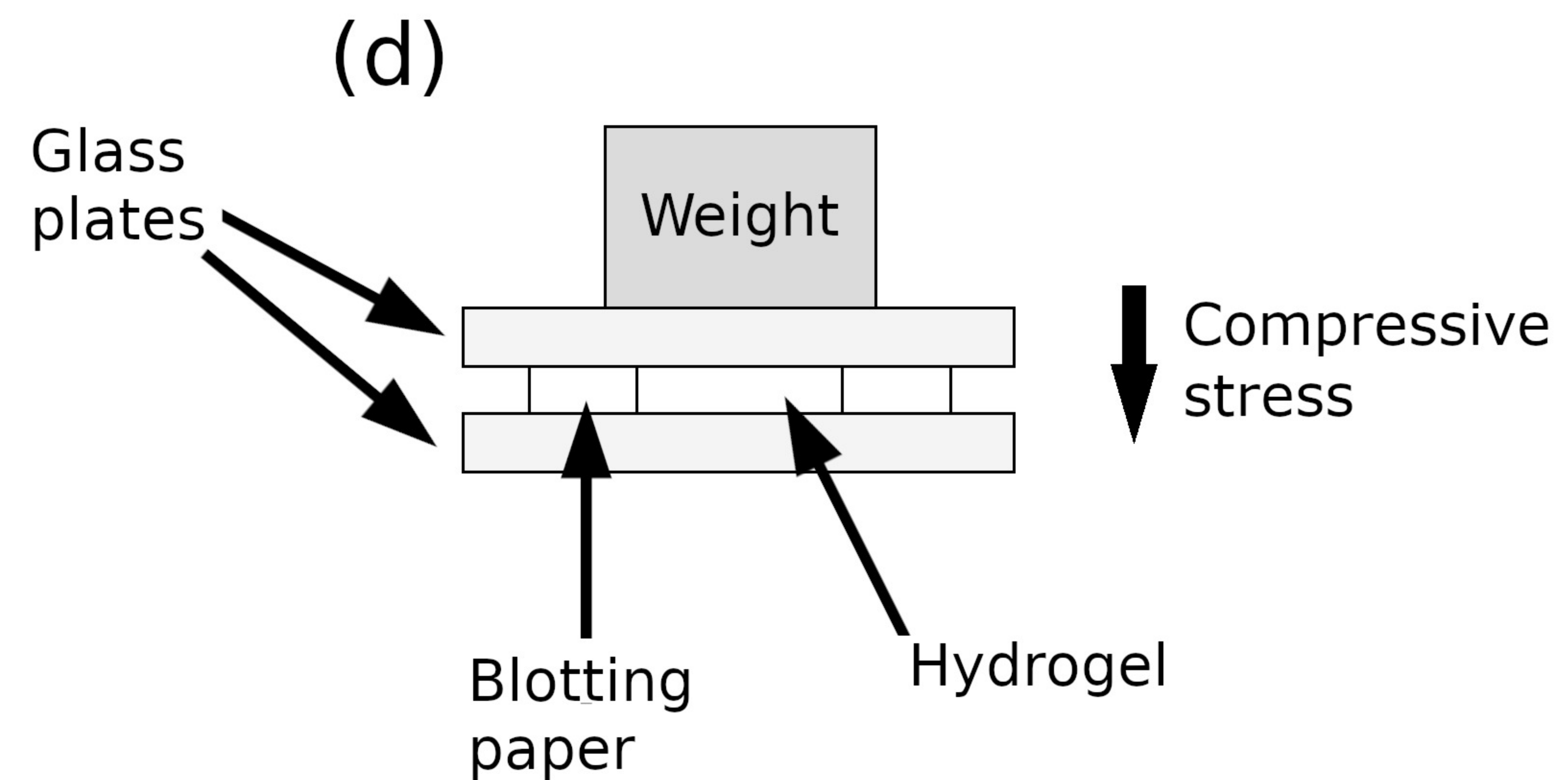

Supplement: Supplementary file 1 [file gels-09-00039-s001.zip › Figures_supplementary/Dehydration-eps-converted-to.pdf]

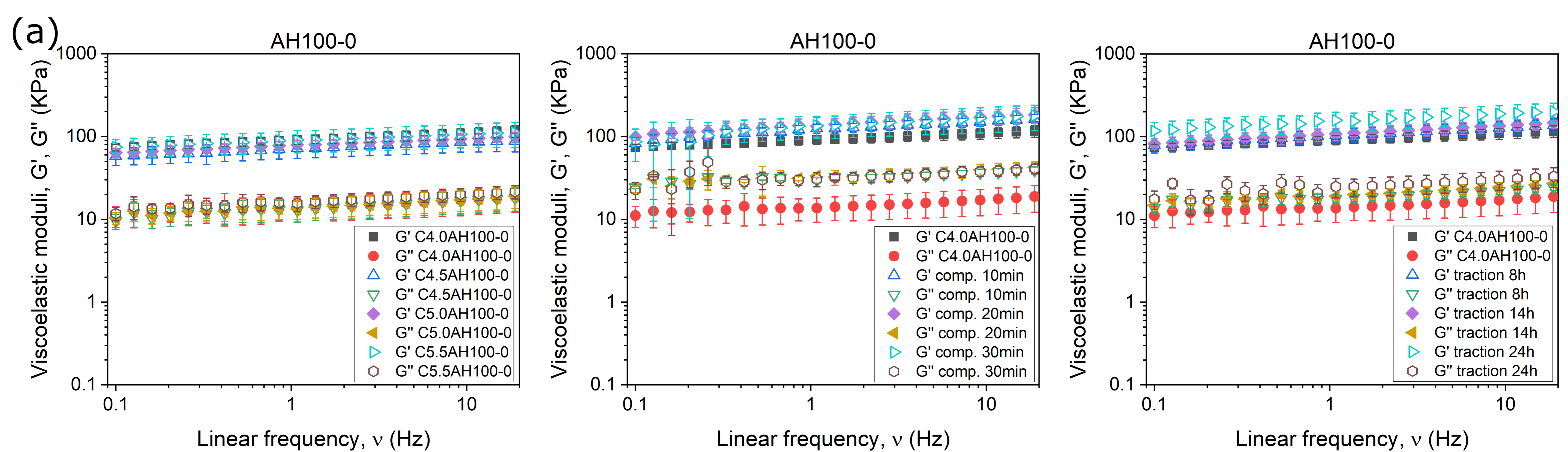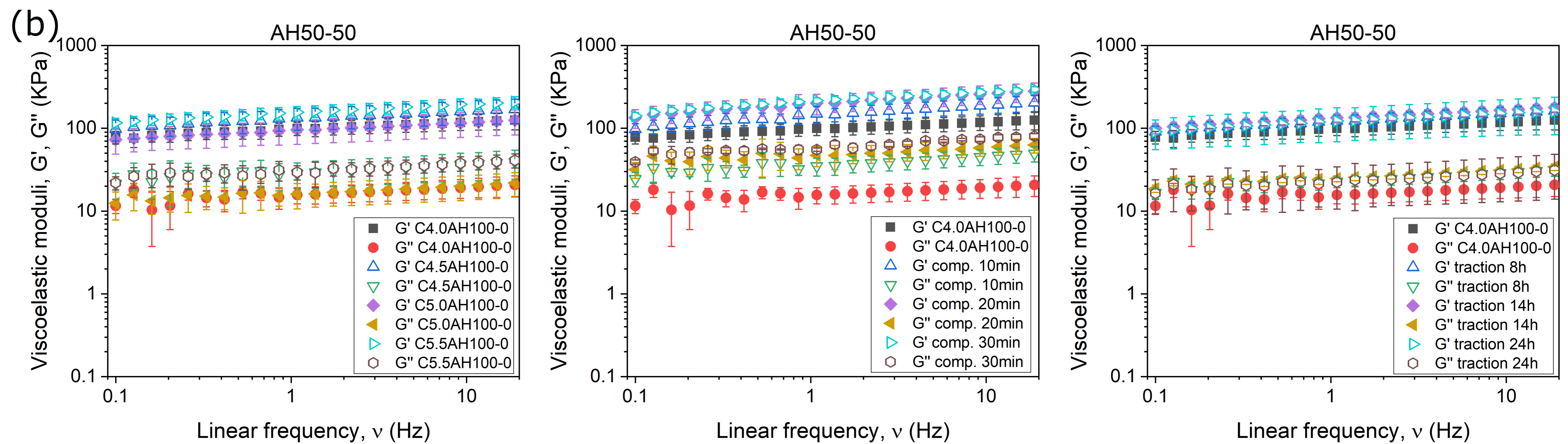

Supplement: Supplementary file 1 [file gels-09-00039-s001.zip › Figures_supplementary/Frequency_sweeps-eps-converted-to.pdf]

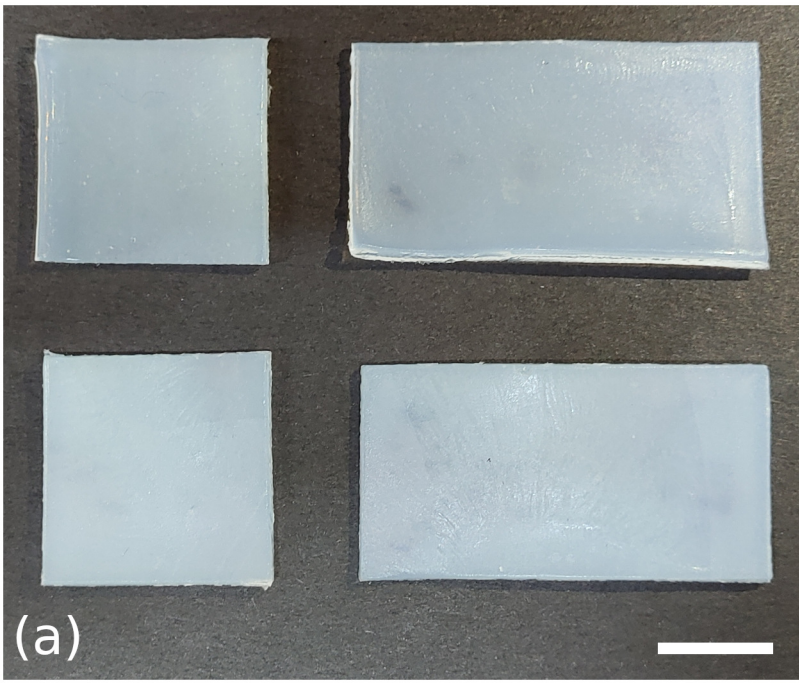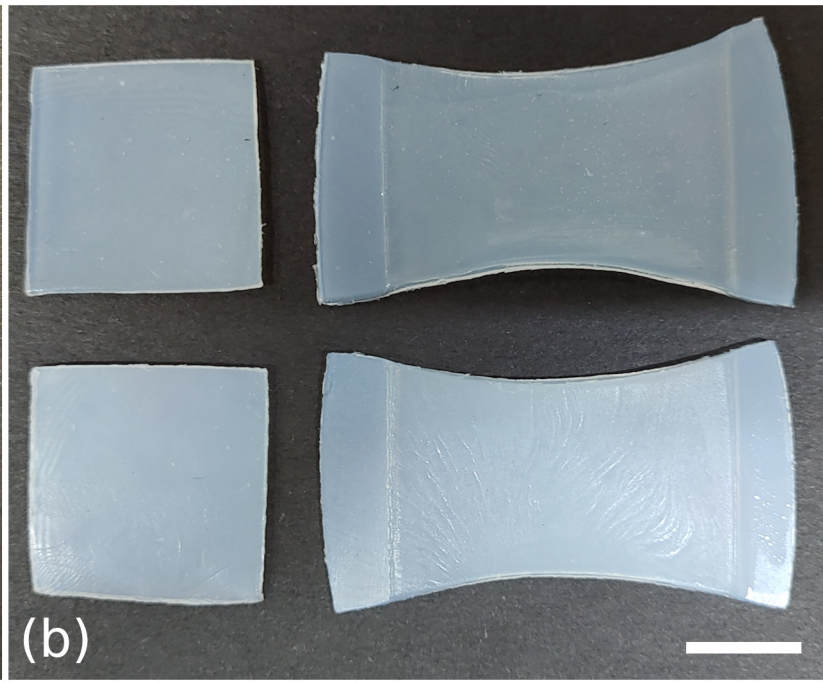

Supplement: Supplementary file 1 [file gels-09-00039-s001.zip › Figures_supplementary/Macroscopic-eps-converted-to.pdf]

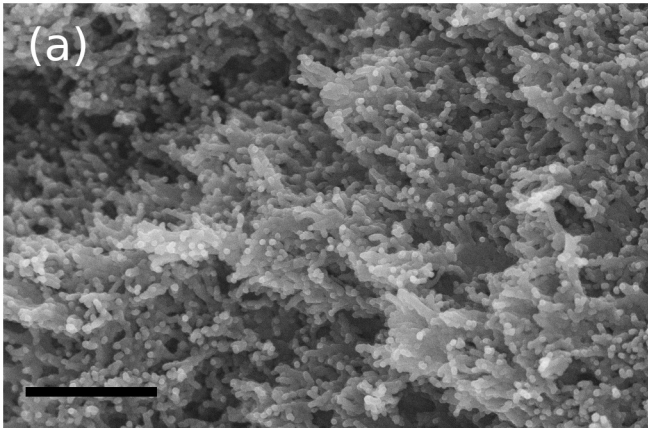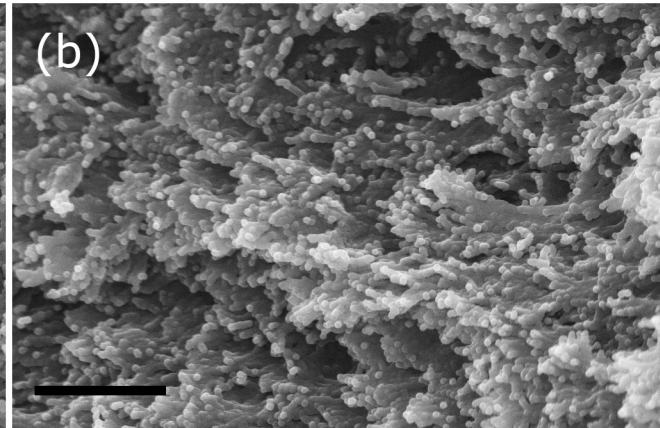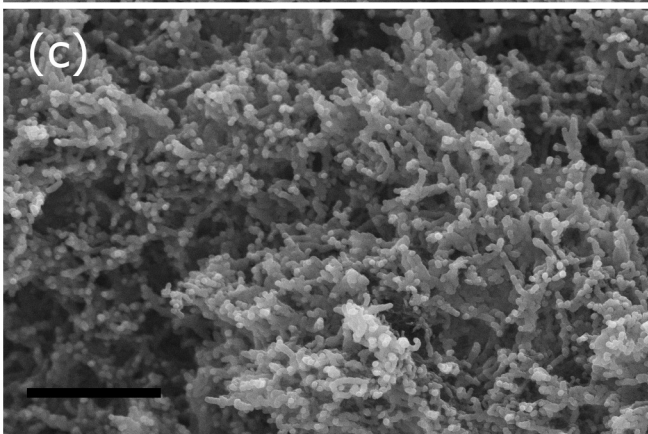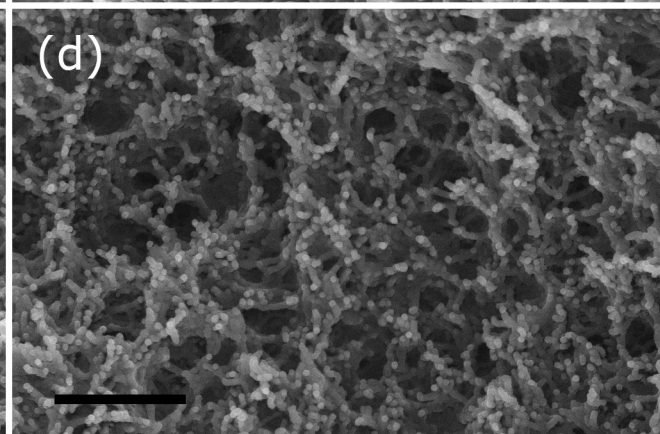

Supplement: Supplementary file 1 [file gels-09-00039-s001.zip › Figures_supplementary/Microscopy_supp-eps-converted-to.pdf]

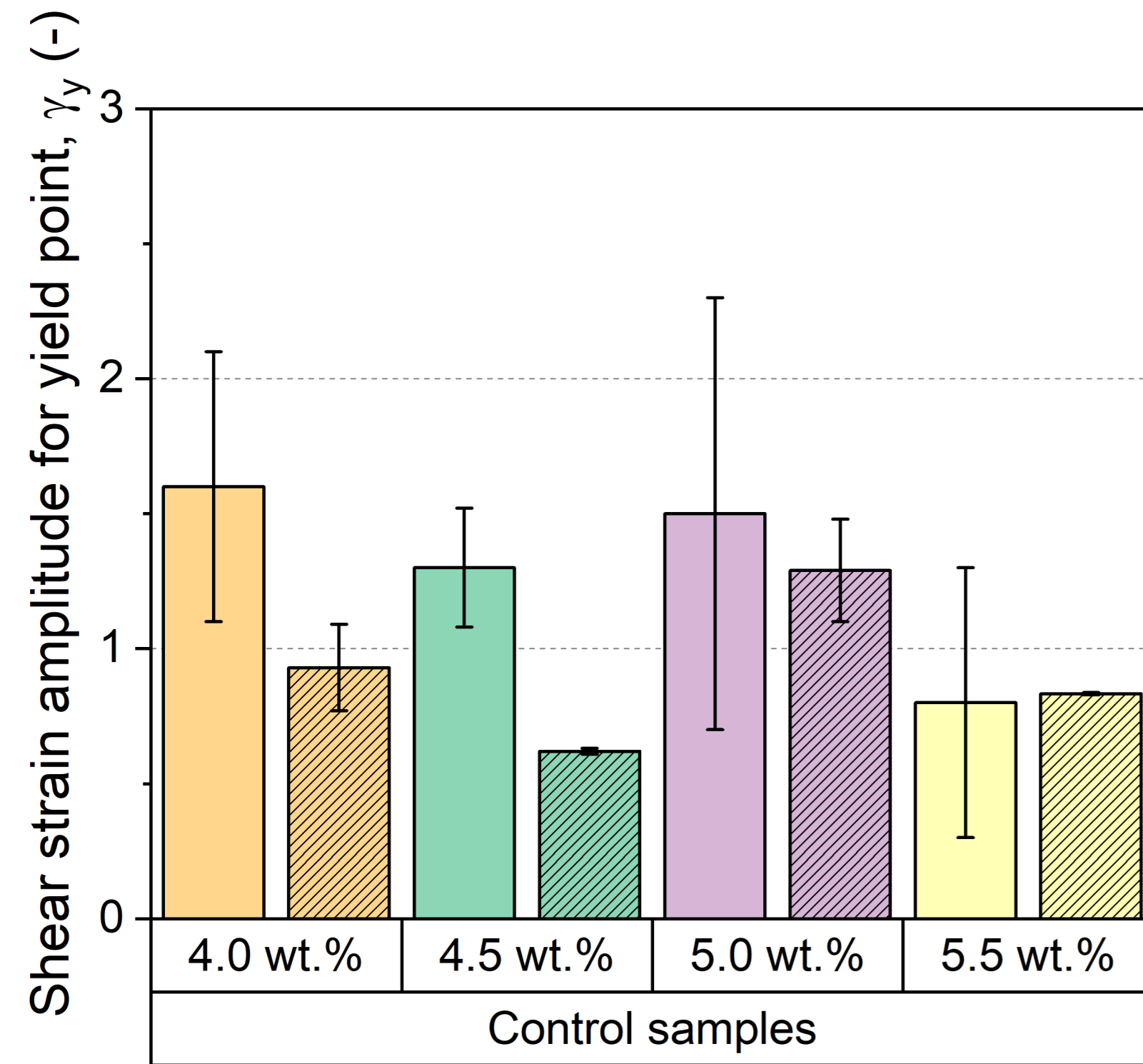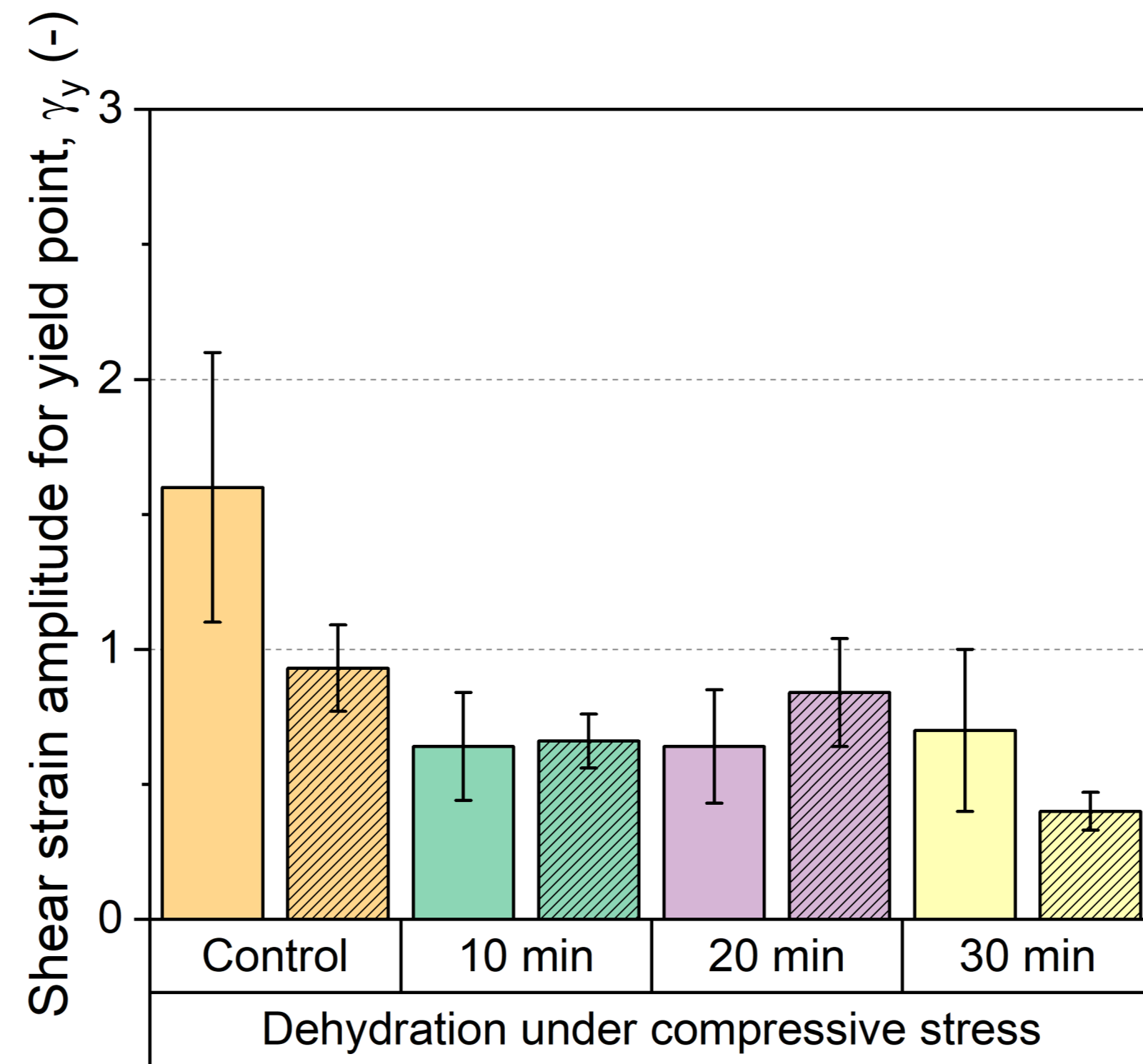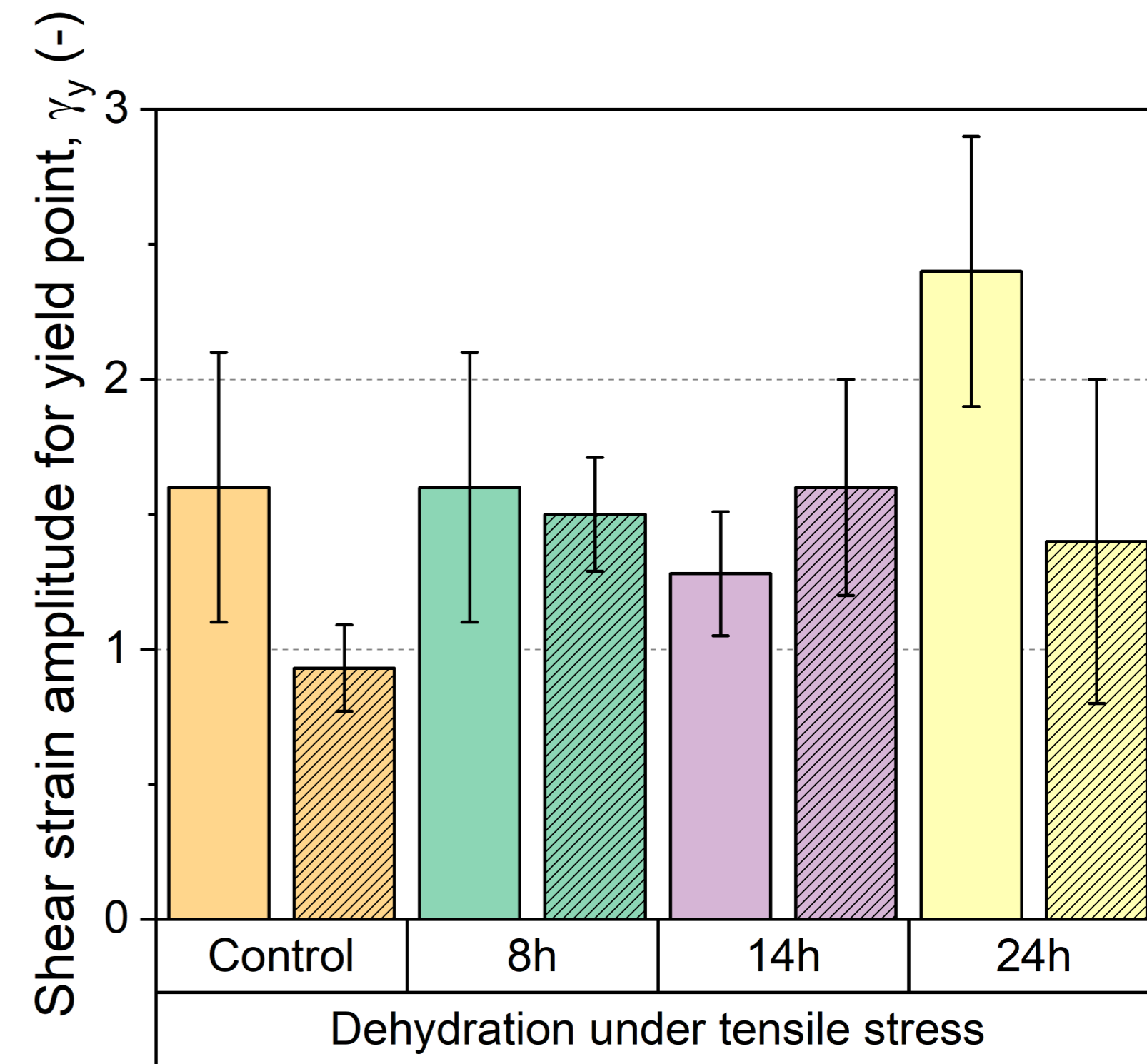

Supplement: Supplementary file 1 [file gels-09-00039-s001.zip › Figures_supplementary/Shear_supp-eps-converted-to.pdf]

(a)

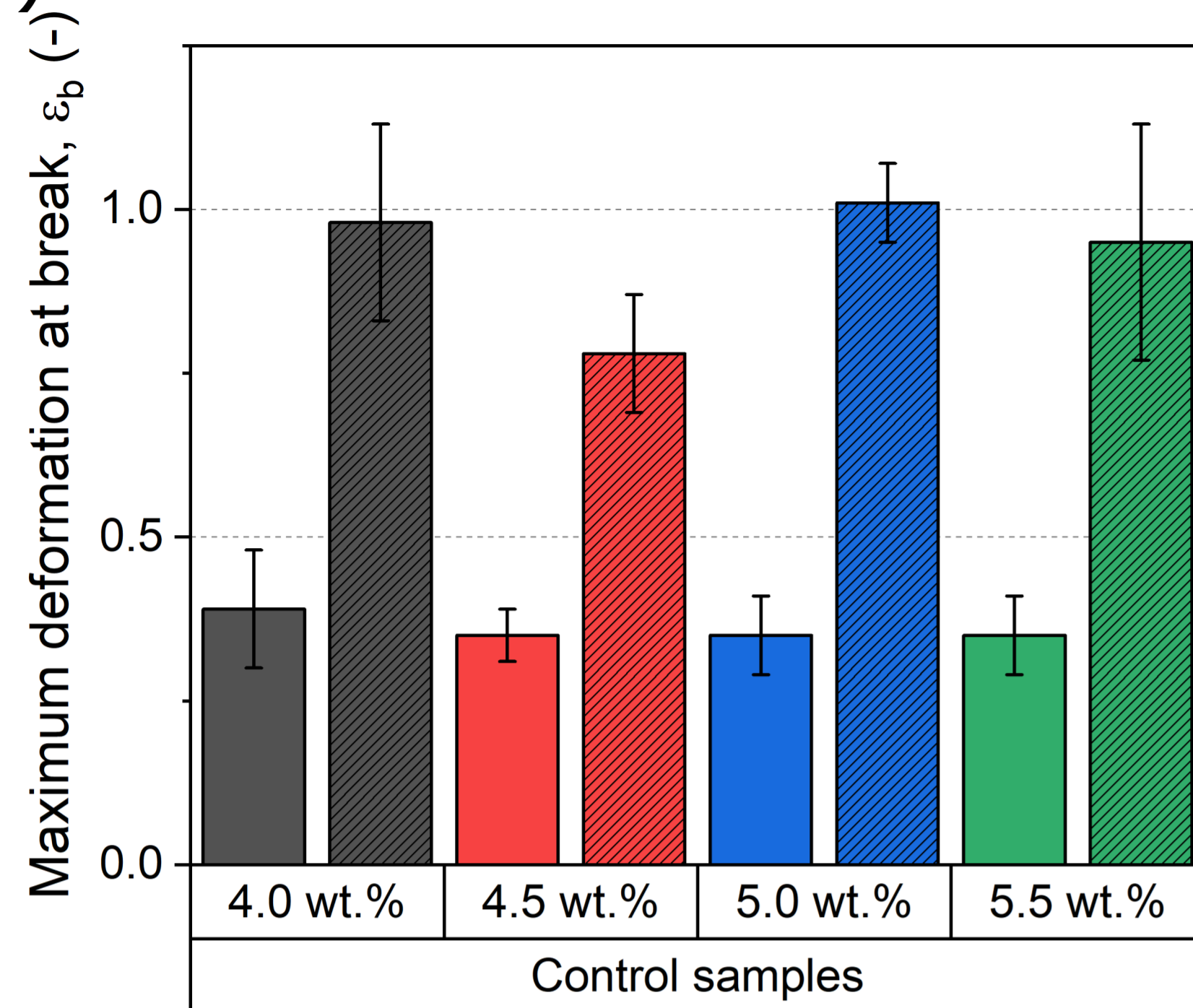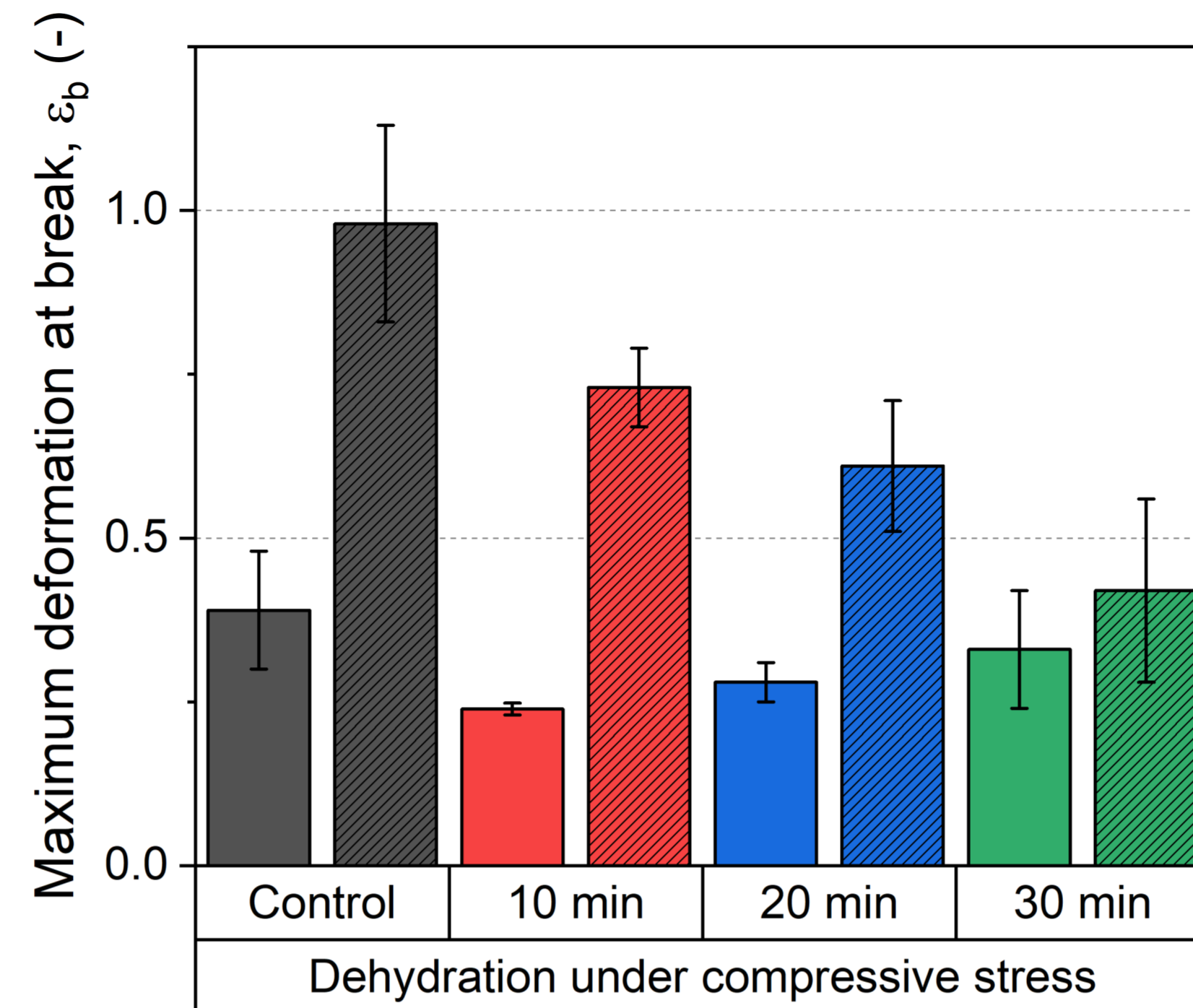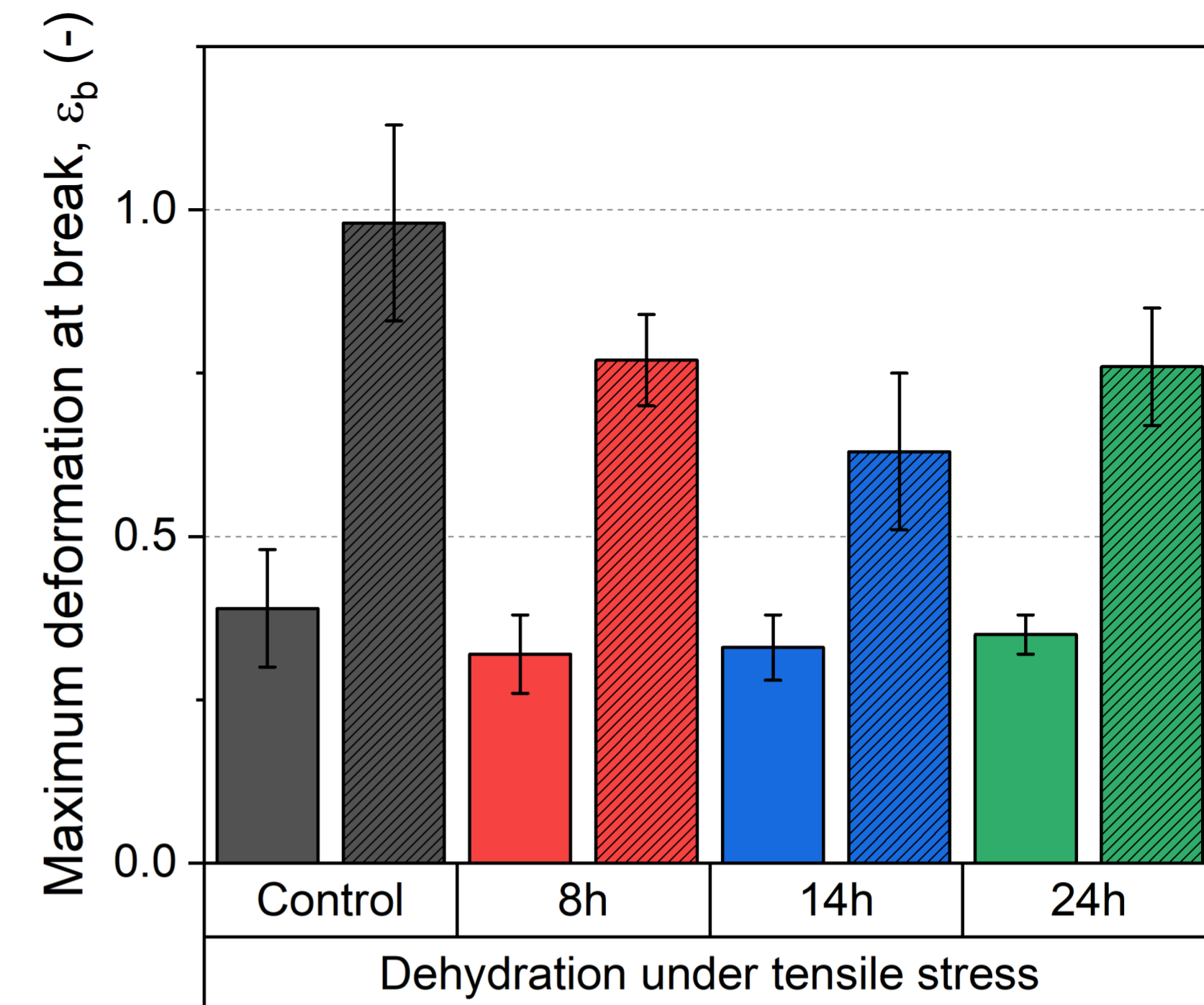

(b)

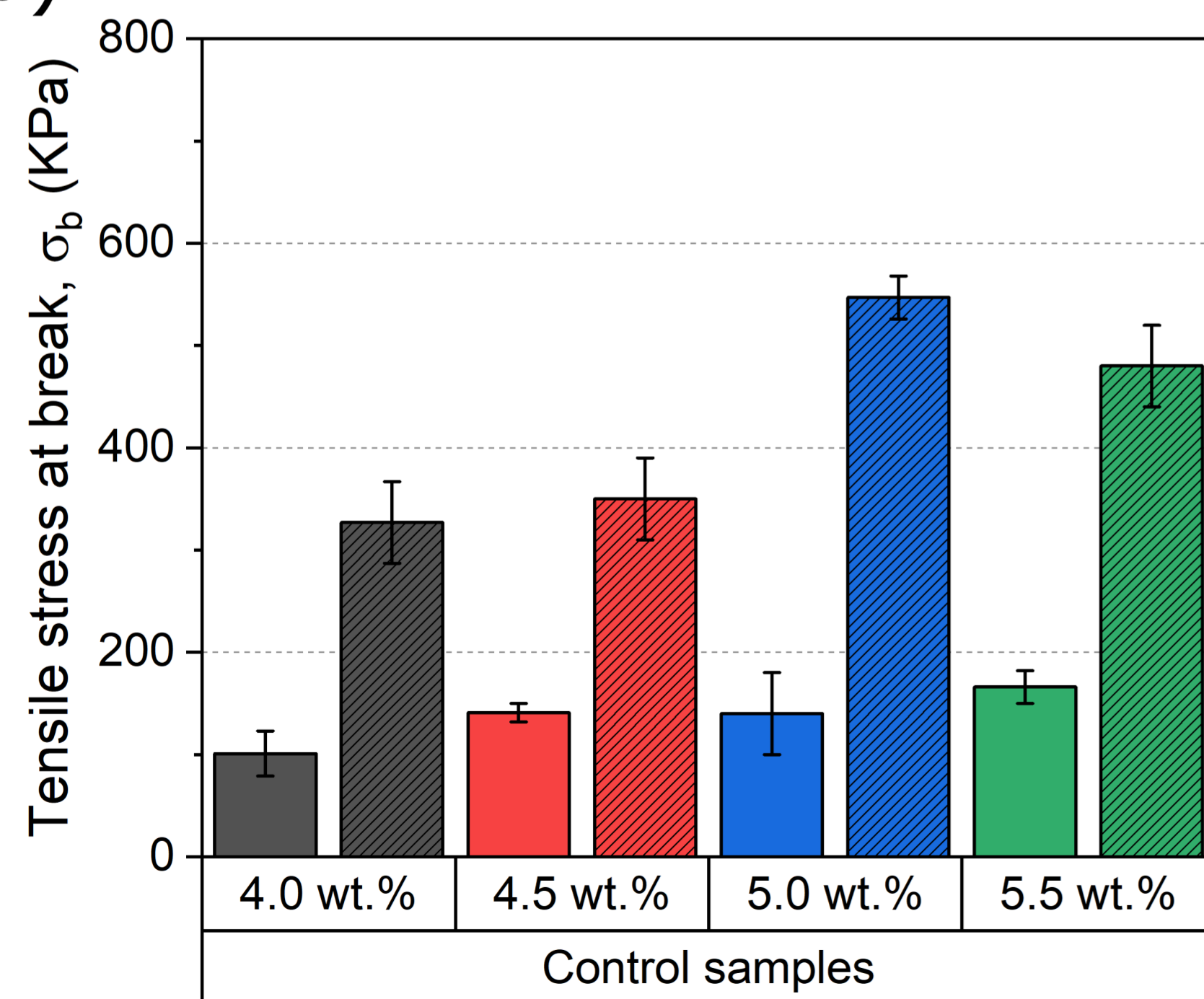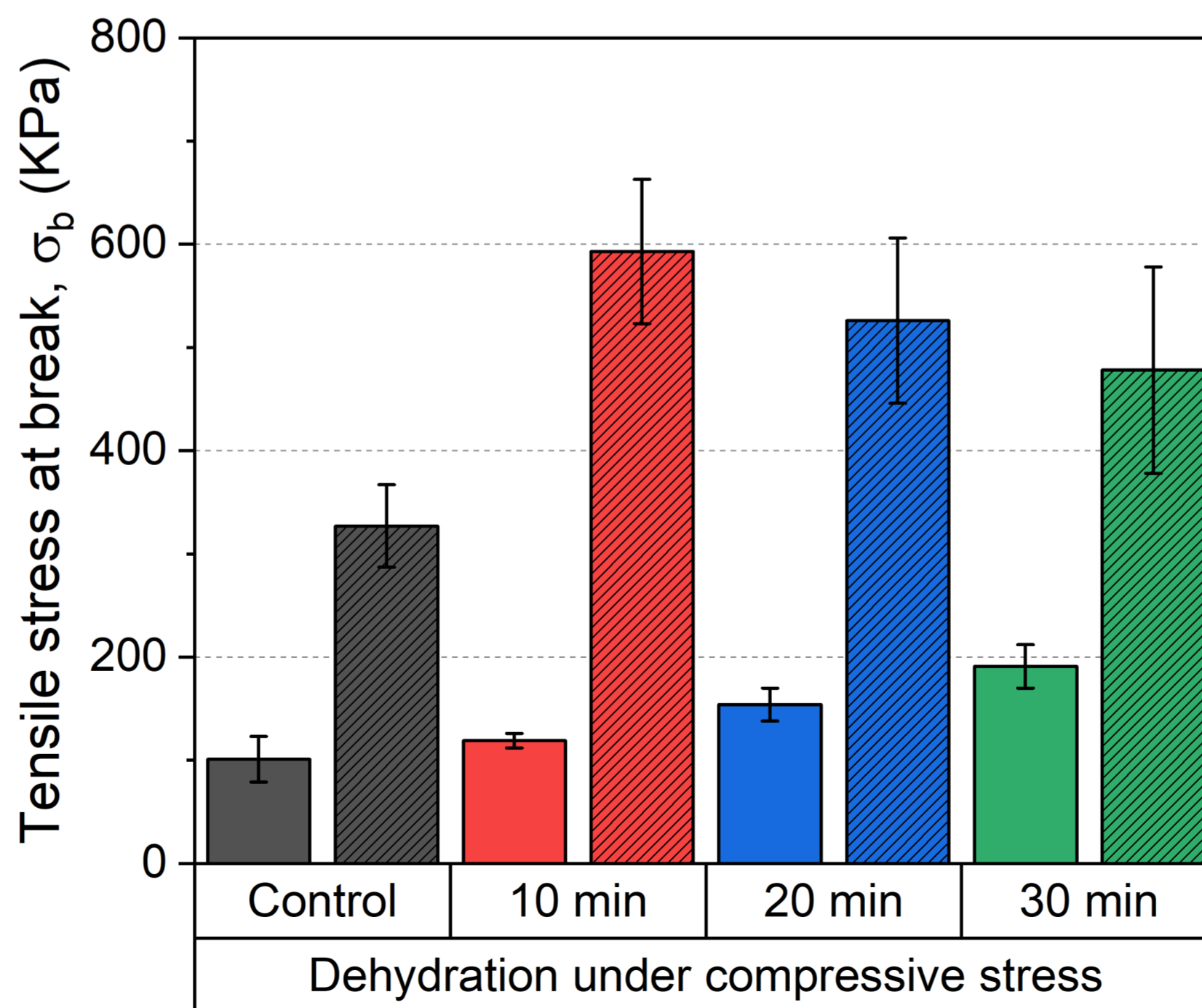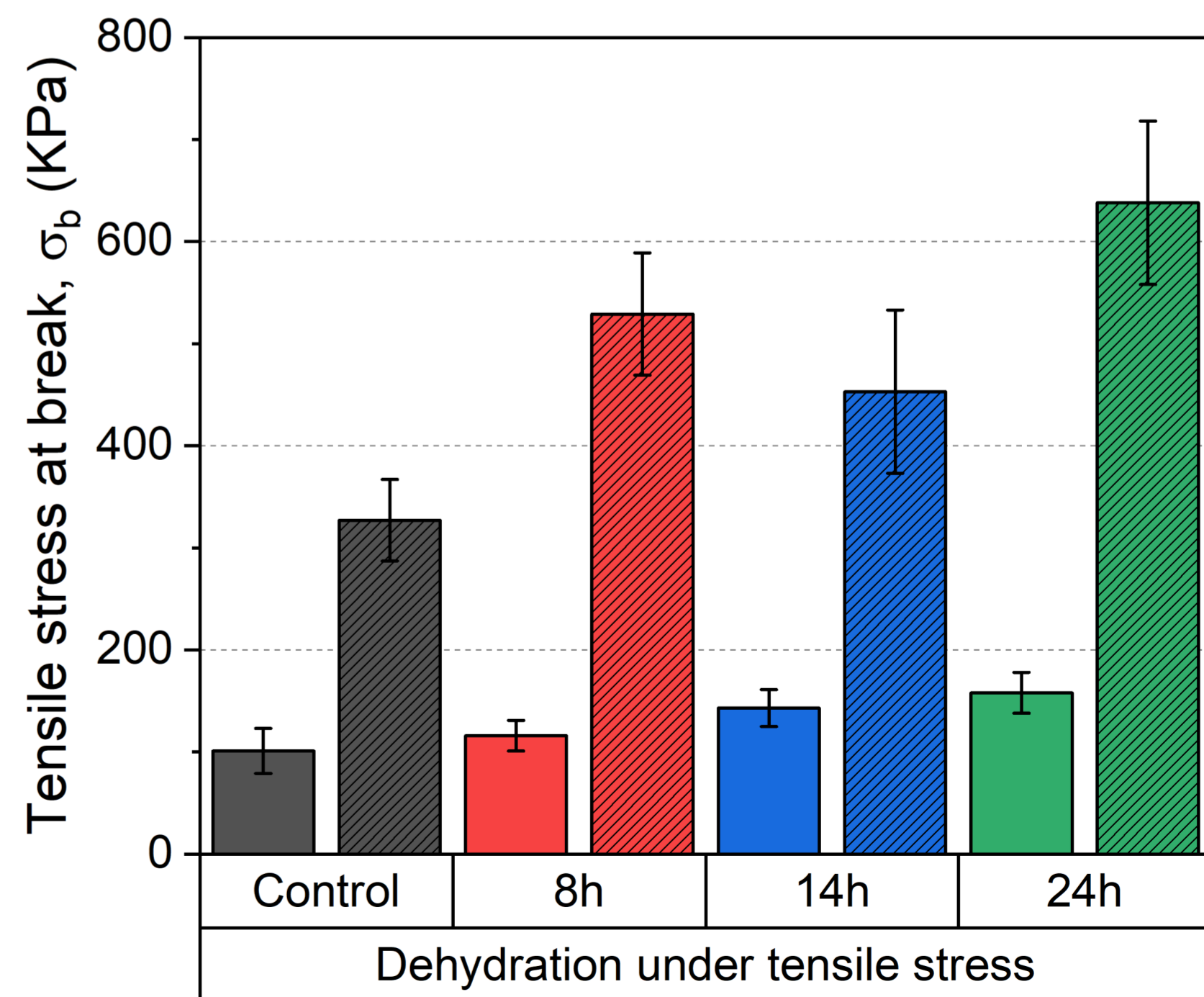

Supplement: Supplementary file 1 [file gels-09-00039-s001.zip › Figures_supplementary/Traction_supp-eps-converted-to.pdf]
